# Large On–Off Enhancement of Au Nanocatalyst Contacts to ZnO Nanowires with Bulk and Surface Oxygen Modification

**DOI:** 10.1021/acsami.4c17872

**Published:** 2025-03-12

**Authors:** Alex M. Lord, Vincent Consonni, Fabrice Donatini, Demie M. Kepaptsoglou, Quentin M. Ramasse, Jon E. Evans, Martin W. Allen, Mark S’ari, Mac Hathaway, Irene M. N. Groot

**Affiliations:** † Centre for NanoHealth, College of Engineering, Swansea University, Swansea SA2 8PP, United Kingdom; ‡ Leiden Institute of Chemistry, Universiteit Leiden, PO Box 9502, Leiden 2300 RA, Netherlands; § CNRS, Grenoble INP, LMGP, Université Grenoble Alpes, F-38000 Grenoble, France; ∥ CNRS, Grenoble INP, Institut NEEL, Université Grenoble Alpes, F-38000 Grenoble, France; ⊥ SuperSTEM Laboratory, SciTech Daresbury Campus, Daresbury WA4 4AD, United Kingdom; # School of Physics, Engineering and Technology, University of York, Heslington, York YO10 5DD, United Kingdom; ¶ School of Chemical and Process Engineering and School of Physics and Astronomy, University of Leeds, Leeds LS2 9JT, United Kingdom; ■ MacDiarmid Institute for Advanced Materials and Nanotechnology, Department of Electrical and Computer Engineering, University of Canterbury, Christchurch 8140, New Zealand; ● School of Chemical and Process Engineering, University of Leeds, Leeds LS2 9JT, United Kingdom; ▲ Harvard Center for Nanoscale Systems, Cambridge, Massachusetts 02138, United States of America

**Keywords:** ZnO, Nanowires, Schottky contacts, Piezotronic applications, Bulk modification, Surface modification

## Abstract

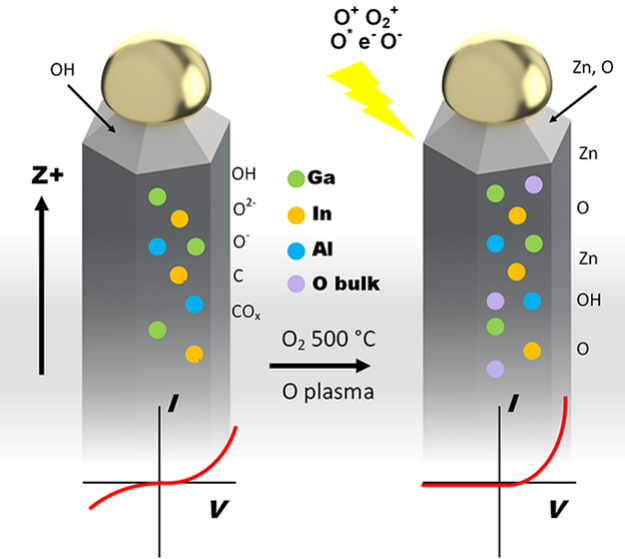

Schottky diodes have been a fundamental component of electrical circuits for many decades, and intense research continues to this day on planar materials with increasingly exotic compounds. With the birth of nanotechnology, a paradigm shift occurred with Schottky contacts proving to be essential for enabling nanodevice inventions and increasing their performance by many orders of magnitude, particularly in the fields of piezotronics and piezoelectric energy harvesting. ZnO nanomaterials have proven to be the most popular materials in those devices as they possess high piezoelectric coefficients, high surface sensitivity, and low resistivity due to the high native n-type doping and low hole concentration. ZnO nanowires grown by vapor phase techniques with the aid of a metal catalyst provide a ready-made epitaxial Schottky contact free from interfacial layers and major defects. We show here with the most comprehensive experimental investigation to-date of Au nanocontacts to ZnO nanowires that the modulation of bulk and surface oxygen can dramatically increase the rectifying quality of these contacts when applied in the metal–semiconductor–metal (M-S-M) device configuration with potential barriers approaching the performance of planar contacts on single crystal ZnO. Before modification, the Au-ZnO nanowire contacts in a rectifying-nanowire-ohmic M-S-M device configuration typically show limited current rectification and electrical transport properties dominated by surface effects and tunneling at the contact edge. Interestingly, the oxygen modulation only has a minor effect on the resistivity as the high-resolution cathodoluminescence spectroscopy shows that the dominant donors are In, Ga, and Al with no visible band emissions often associated with detrimental point defects. The spectroscopy also revealed that carbon is incorporated into the bulk that may present interesting magnetic properties for future spintronics applications. Atomic-resolution electron microscopy confirms the Zn-polar orientation of the high-quality single crystal nanowires used for the electrical measurements. X-ray photoelectron spectroscopy shows oxygen-annealed nanowires have fewer surface oxygen defects, and when that difference is coupled with a reduction in surface oxygen vacancies via oxygen plasma treatment, the current rectification can increase by several orders of magnitude with a much lower dispersion in the effective potential barrier properties when compared to those that are not annealed. This study concludes after the electrical measurements of 66 nanowire contacts/M-S-M structures with diameters as small as 25 nm using a scanning tunneling microscopy probe that effective device potential barrier heights of 0.65 eV and on–off ratios of 3 orders of magnitude can be achieved. Interestingly, this change in contact properties is transient in nature, revealing dynamic surface effects can govern the rectifying behavior and surface passivation techniques are desirable to achieve consistent performance. This work shows the overriding effects of surface defects and adsorbates on the sloping facets near the Au contact edge and the potential for this effect to be used to control the electrical transport properties and produce molecular-scale sensors to greatly enhance the performance of many piezotronic and energy harvesting devices.

## 1 Introduction

Over the last two decades ZnO nanomaterials have been at the center of the burgeoning fields of energy harvesting nanogenerators and piezotronics.^1−3^ Controllable electrical contacts are essential if reproducible and optimized devices are to be achieved in these fields.^4^ On single crystal ZnO, point defects, particularly oxygen vacancies, have been shown to have an overriding effect on Schottky contact properties.^5,6^ Processing steps, before and after metal deposition, have been developed that reduce the concentration of point defects at or near the interface with many focusing on oxidizing treatments.^7^ Oxygen plasma treatment of the bare ZnO surface and oxidizing the metal during deposition have both been shown to greatly improve the Schottky barrier height, ideality factor, and reverse bias leakage.^8,9^ However, there are only a limited number of examples manipulating the oxygen content of ZnO nanomaterials with the intention of optimizing Schottky contacts.^10,11^ Even more so, there are very few examples for nanowires (NWs) with diameters under 50 nm with studies focusing on surface reactions to increase device performance.^12,13^ When ZnO NWs are reduced in size to diameters as small as 25 nm, there appear to be no works aiming to fully assess rectifying electrical contacts in the typical metal–semiconductor–metal (M-S-M) device configuration by tuning the bulk and surface oxygen. Part of the problem is the difficulty in experimentally measuring and characterizing the properties of nanosized contacts while dealing with high surface variability and sensitivity to environmental factors of the ZnO NWs. Our previous work showed that the metal catalyst growth particles at the tips of ZnO NWs after vapor phase growth can provide rectifying contacts but due to edge tunnelling effects rectification is limited to an on–off ratio of ∼10.^14^ For large scale contacts, this would be regarded as very weak rectification and makes simple current–voltage data analysis with typical transport relationships inappropriate. In this work, we investigate the role of oxygen in Au nanocatalyst contacts to ZnO NWs with end-of-growth oxygen modulation and postgrowth oxygen plasma treatment. In many of the NWs the oxygen plasma treatment is shown to create a large enhancement of the on–off performance of the Au-ZnO nanocontacts in the M-S-M configuration measured using a single scanning tunneling microscopy (STM) probe. We show increases in rectification by 2–3 orders of magnitude and the removal of any indication that edge effects dominate electrical transport across the Au-ZnO interfaces. To achieve a broad understanding of the materials and rule out potential contributing factors, we provide a comprehensive analysis with cathodoluminescence spectroscopy (CL) to reveal that the dominant donors are In, Ga, and Al. X-ray photoelectron spectroscopy (XPS) provides an understanding of the surface, with the most influential effect being the reduction of emissions associated with oxygen vacancies after both oxygen annealing and oxygen plasma. Atomic-resolution scanning transmission electron microscopy (STEM) imaging and spectroscopy are applied to assess the structure, composition, and crystal polarity showing the NWs grow with Zn-polarity and with no major defects and abrupt epitaxial interfaces with the Au contacts on the (0001) ZnO top facet. However, even with this great enhancement in rectifying capabilities, the electrical measurements show the on–off enhancement can be transient leading to the conclusion that dynamic surface species and defect migration/surface reconstruction, particularly on the ZnO facets near the Au interface, can have an overriding effect on the exact nature of the enhanced contacts.

## 2 Materials and Methods

### 2.1 Nanowire (NW) Growth

ZnO NWs were grown on a-plane Al_2_O_3_ with the aid of a sputtered Au ∼ 5 nm film using the standard carbothermal method at 900 °C, 30 mbar, 49 sccm Ar and 1 sccm O_2_ for 2 h using a powder ZnO and carbon source.^15,16^ Growth proceeded for 2 h, the gas flow was switched off once the reactor had cooled to 850 °C and the growth chamber was pumped at a pressure of 0.1 mbar until reaching room temperature (RT).

### 2.2 Oxygen Modulation with Annealing and Plasma Treatment

The NWs that were grown at 900 °C and naturally cooled to RT in the tube furnace were used for one sample type (identified later as HoD). Other samples were grown using the same method, with one modification at the end of the growth process. On cooling to 500 °C in the tube furnace at 0.1 mbar the temperature was then maintained for 1 h and a constant flow of O_2_ at a pressure of 3 mbar was introduced to produce oxygen-annealed NWs (later identified as LoD). Several of these samples were used to optimize the subsequent oxygen plasma treatment to achieve the greatest change in current–voltage characteristics (increase in current rectification) with plasma treatments ranging from 40 s to 10 min. Using a standard desktop plasma reactor typically used for sample cleaning the individual samples were pumped down to ∼10^–3^ mbar for 10 min before O_2_ was introduced and a treatment for 4 min at 50 W and ∼0.2 mbar was found to produce the greatest rectification of the *I*–*V* characteristics at ±1 V. Samples were immediately loaded into the multiprobe STM/CL/XPS chamber and evacuated immediately after plasma treatment to minimize exposure to atmospheric effects.

### 2.3 Electrical Measurements Methods

#### 2.3.1 Multiprobe Scanning Tunneling Microscopy (STM) Electrical Measurements

ZnO NWs from the two different “end-of-growth” process variations were electrically measured using a single scanning tunneling microscopy (STM) tip in an Omicron LT Nanoprobe after they had been mechanically transferred to Au TEM half-grids. Au grids and sample measurement plates were cleaned with oxygen plasma at 100 W for 15 min before cutting the Au grid in half (exposing unmodified Au) and NWs were mechanically transferred in a directional manner to the outermost fingers with the Au catalyst tips pointing outward.^17,18^ NWs in the array configuration and on the grids were screened with a Hitachi S4800 SEM instrument using the secondary electron and backscattered electron detectors. The work here expands on previous work by changing standard Cu grids for Au grids, which allows chemical modification of the NWs and cleaning of the grids without chemical reactions (such as oxide formation) that could create a Schottky barrier or insulating layer at the NW-grid contact. The NW-grid contact was tested on several NWs by placing the measurement tip on the side facet of the NWs and measuring the *I*–*V* characteristics, which proved to be linear with a current magnitude similar to the measurements on the Au catalyst contacts. After the grid samples were loaded into the UHV (10^–11^ mbar) instrument the glass ports were covered such that the sample was in complete darkness. The initial electrical measurements on “as-grown” NWs were conducted several days after loading. The STM tungsten tip was annealed immediately before each round of electrical measurement on each sample and each sample treatment to remove oxides and contamination.^19^ The tip was positioned with the aid of *in situ* SEM imaging, and this was blanked once tip contact had been made to each NW. The additional electron beam induced current is always observed to decay within a few seconds of blanking the beam. Once the electrical measurements of the NWs in the as-grown state were completed, samples were removed from the UHV chamber and immediately plasma treated and then loaded back into the UHV instrument. Measurements on the plasma treated NWs were conducted ∼24 h after loading to allow photoexcited carriers to relax and then over a period of a further 48 h to monitor transient effects on the *I*–*V* characteristics. NWs were then analyzed with a FEI Titan Themis 300 with bright-field (BF) imaging and energy dispersive X-ray spectroscopy (EDX).

#### 2.3.2 Errors Influencing the Interpretation of the Electrical Measurements

Errors in measured values, such as rectification ratio, ideality factor, and resistance, are the standard deviation of measured values. Errors in NW diameters are the range from the 25th to the 75th percentile of a Gaussian tail approximation of the SEM edge resolution and the reported values are from 50% edge intensity. The error in diameters is used to calculate the maximum and minimum values for R_Au_ (the ratio of the Au diameter to NW diameter) that are reported here. NWs that were inspected with transmission electron microscopy (TEM)/ scanning-TEM (STEM) were determined to have error in diameter measurement of ±0.75 nm from line profiles that reduces the error in R_Au_ and is displayed by the error bars of those NWs.

Error in resistivity and current density stem from error in the diameter measurements, and the reported values are the average range of the maximum and minimum of calculated values for each NW. Measurement error in current density propagates to the calculation of effective barrier height; however, the error this introduces into the calculated values is ∼1% of the calculated value so these error bars are omitted. The variation in ideality factor measurements in some cases was ∼10% on each NW and is reported here.

### 2.4 Aberration-Corrected Scanning Transmission Electron Microscopy (STEM)

High-resolution high angle annular dark field (HAADF) and BF imaging was carried out in a Nion UltraSTEM100 scanning transmission electron microscope (STEM) operated at 100 keV primary beam energy that was equipped with a UHV Enfina EELS spectrometer. The probe-forming optics, corrected for aberrations up to fifth order, were configured to provide ∼50 pA of beam current with a 31 mrad beam convergence semiangle, for an estimated probe size of 0.8 Å. The inner and outer radii of the HAADF detector were calibrated at 79 and 195 mrad, respectively, and of the MAADF at 40 and 86 mrad, respectively (when used simultaneously with the HAADF). Using this technique, NWs were tilted to an available zone axis of the NW or Au particle, and the structure was imaged using phase contrast and HAADF imaging. Image simulations were performed with QSTEM using parameters reflecting the experimental conditions.^20^ To ensure minimal beam damage of the structures, core-loss EELS was carried out after initial imaging and electrical assessment. Compositional EELS mapping was performed using the same beam configuration using a 100 keV primary beam energy and an exposure of 0.06 s per pixel. Chemical maps were created by integrating over a suitable energy window the intensity above the relevant EELS edges (Zn-L_2,3_, O–K, and C–K) after removal of the decaying background using a power law model. The EELS data was systematically denoised using principal component analysis.

### 2.5 Cathodoluminescence (CL) at 5K

Five K CL measurements of single NWs located in each of the growth arrays (ZnO NWs with and without oxygen annealing and before and after oxygen plasma treatment) with the Au contact on their tip were performed using an FEI Inspect F50 FESEM equipped with a liquid helium cooled stage. The CL signal was collected through a parabolic mirror and analyzed with a 550 mm focal length monochromator equipped with 600 grooves/mm diffraction grating. CL spectra were recorded with a thermoelectrically cooled silicon CCD detector. The low acceleration voltage of 7 kV and very small spot size (i.e., less than 10 nm) were used to create the CL signal at the center of the uppermost NW tip close to the interface with the 30 nm thick Au contact. By considering the penetration depth of the electron beam using Casino software, we found that the CL intensity mostly originated from a depth of ∼220 nm from the ZnO NW tip below the Au contact. The electron beam current was several pA. NWs were analyzed by CL measurements before and after oxygen plasma treatment in the top-down configuration probing through the Au contact and additionally in the profile configuration through the ZnO sidewall surface with no distinguishable difference detectable between the two analysis configurations. To make a direct comparison of the different collected 5 K CL spectra, the measurements were performed during the same run of experiments, with the four samples placed on a dedicated copper holder. Simulations of the excitation depth were performed with the CASINO software.

### 2.6 X-ray Photoelectron Spectroscopy (XPS)

#### 2.6.1 XPS Experimental Method

The arrays of vertical NWs were analyzed with and without oxygen annealing and before and after oxygen plasma using a Kratos Axis Ultra-DLD photoelectron spectrometer, utilizing monochromatic Al Kα radiation, operating at 144 W (12 mA x 12 kV) with an effective energy resolution ∼400 meV and a takeoff angle of 90°. Charge compensation was achieved utilizing the Kratos magnetic lens system. Survey spectra were collected at a pass energy of 160 eV, from −5 to 1200 eV, while high-resolution spectra were collected between 520 and 540 eV (O 1s), from 1012 to 1030 eV (Zn 2p 3/2), from 276 to 300 eV (C 1s). For each scan, a pass energy of 40 eV was used. Care was taken to ensure that no signal was detected from the chamber or sample holder (e.g., Al 2p, Fe 2p3/2), particularly as multiple locations were measured for each sample. Although the slot aperture was used to collect electrons to maximize signal (700um x 300um), samples measured at least 5 × 5 mm and measurements were not taken from near the sample edges. The pressure was 10^–9^ mbar in the analysis chamber. Energy calibration was performed before XPS analysis on a standard Ag sample to determine the Fermi edge. Both samples (with and without oxygen annealing) were analyzed then removed from the XPS instrument and treated with oxygen plasma *ex situ* for 4 min at 50W and pressure 0.1 mbar before further analysis with XPS at 12-h intervals from loading until 48 h at three locations on each sample.

#### 2.6.2 XPS Data Analysis

The XPS data was analyzed with CasaXPS^21^ and for these samples the Zn 2p3/2 peak was fitted with a single component on a Tougaard background with no constraints. The O 1s peak was fitted on a Shirley background with three components corresponding to the O_Zn_ lattice oxygen, O_S1_ associated with oxygen ions, vacancies, and hydroxyls and the O_S2_ peak associated with water or carbon molecules. The O_Zn_ peak was fixed with a full width at half-maximum (fwhm) of 1.15 eV obtained from the fitting of the as-grown samples while the O_S1_ peak was constrained with a fwhm ≤1.9 eV and fixed at a binding energy (BE) of 1.5 eV greater than O_Zn_. O_S2_ was not constrained in fwhm but was fixed to a BE of 3.4 eV higher than that of O_Zn_. Removing these constraints yielded proportionally similar results for peak positions, fwhm, and area when comparing samples. Peaks were constrained in this manner according to accepted peak positions to capture the weak but increasing O_S2_ component that the XPS analysis showed over time after plasma treatment. The C 1s peak was fitted with three components on a Tougaard background with the fwhm of the higher BE peaks constrained to the same fwhm of the main peak. The three C 1s components were found to increase with time after plasma treatment within the XPS chamber (10^–9^ mbar).

### 2.7 Photoluminescence (PL) Spectroscopy at 4K

PL spectroscopy was performed at 4 K on “as-grown” ZnO NW arrays in a reduced pressure helium flow cryostat (Oxford Optistat) to provide detailed information on the excitations at the near band edge (NBE). Samples were mounted onto an aluminum sample holder using silver colloidal paste and excited in a near-backscattering geometry (E ⊥ c polarization) by the focused beam of the 325 nm line of a 15 mW HeCd laser. A Jobin-Yvon 1000 M spectrometer with a focal length of 100 cm and a 1200-line/mm holographic grating was used to disperse the incoming PL emission with a cooled Hamamatsu R943–02 photomultiplier tube operating in photon counting mode for detection. Spectral resolution exceeded 100 μeV at the operating spectrometer slit widths. The sampling step size was chosen to be 50 μeV for most of the NBE region, with finer steps of 10 μeV for the donor-bound excitons. The well-known sharp atomic transitions of a low-pressure Hg lamp were used for wavelength calibration.

### 2.8 Atomic-Layer Deposition (ALD)

Deposition of Al_2_O_3_ was performed in a Savannah 200 ALD system with 30 cycles at 150 and 250 °C to analyze the layer uniformity, thickness, and NW surface reaction to the processing.

## 3 Results and Discussion

### 3.1 Electrical Measurements of ZnO NWs with Au Nanocatalyst Contacts

NWs from two growth arrays were transferred to Au TEM grids for measurement in the multiprobe STM instrument in a M-S-M configuration typical of single NW piezotronic devices with no lower substrate support and relying on the Au nanocatalyst growth particle as the rectifying contact.^17^ The two NW array samples were grown in exactly the same fashion except one sample was cooled to RT under vacuum while the other sample was annealed in a 3 mbar oxygen atmosphere for 1 h at ∼500 °C during the cooling process, designated as HoD and LoD, respectively, because of the high- and low-concentrations of bulk oxygen point defects that can be expected to exist in the NWs from each sample. Each sample was screened with backscattered electron imaging to identify NWs with the Au particle pointing away from the measurement grid. SEM or TEM images were used to measure the width of the Au particle, and NW that were used to calculate the ratio,
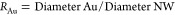
1

Current–voltage (*I*–*V*) measurements were performed at ±1 V on ∼44 HoD NWs and ∼22 LoD NWs. Rectification ratio (RR) is calculated from the current magnitude at +1 V and −1 V and is often used as a “figure of merit” when assessing rectifying contacts, also known as the on–off ratio.

**Figure 1:**
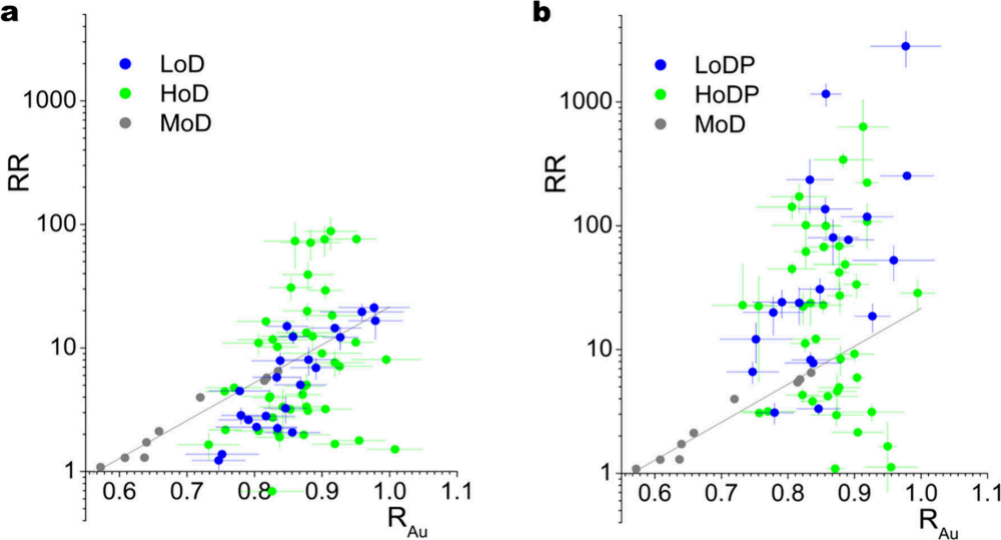
Graphs showing the rectification ratio (RR) plotted against the Au/NW diameter ratio R_Au_: (a) before and (b) after oxygen plasma treatment of the oxygen-annealed (green) and oxygen-deficient (blue) NWs. The gray points (MoD) are measurements from previous work fitted with an exponential trend line.^14^ For greater clarity, data are shown without error bars in Supporting Figure S1.

Figure 1a shows the RR in relation to R_Au_ for each of the HoD and LoD NWs with R_Au_ in the range of 0.7 and 1 and defined by eq 1. Also shown are the data from our previous work that measured NWs with R_Au_ up to ∼0.8, denoted MoD, that underwent a different cooling procedure, namely, cooling to RT at a constant 30 mbar chamber pressure with 2% O_2_ and fitted here with an exponential relationship. Figure 1b shows the same HoD and LoD NWs after a 4 min 50 W oxygen plasma treatment and measured ∼24 h later and these NWs are designated HoDP and LoDP. The oxygen plasma treatment was optimized on a large number of similarly grown samples that tested plasma treatments in the range of 30 s to 10 min. It was found that short treatments (30–120 s) or longer than 4 min considerably decreased the NW resistivity while presenting Ohmic or weakly rectifying *I*–*V* behavior. We also performed similar treatments in an ICP etcher reactor that allowed preprocess chamber conditioning to ensure chamber conditions and residual contamination were not the overriding influence of the plasma treatment on the materials. Clearly, the optimized oxygen plasma treatment has a transformative effect on the rectifying behavior of the NW M-S-M structures with many displaying rectification of several orders of magnitude, particularly the LoDP NWs even at the low biases employed here of ±1 V (Supporting Information Figure S2 shows example linear and log–linear *I*–*V* data for an HoD and LoD NW before and after plasma treatment). The edge-tunnelling dominated electrical transport behavior indicated by the gray line from our previous work has been lost with no discernible relationship with R_Au_ for either plasma-treated sample. A considerable fraction of HoDP contacts remain Ohmic-like after the plasma treatment indicating acceptor plasma-induced surface states are not the dominant influence on the electrical transport behavior of those NWs. To simplify the discussions, we can designate the NW M-S-M structures below the gray line as Ohmic-like and above the line as Schottky-like.

Coppa et al. showed that the presence of oxygen is necessary when cooling ZnO single crystal surfaces to RT to establish an oxygen terminated surface that can lead to upward band bending at the single crystal surface.^22^ However, direct comparison to single crystal surfaces is difficult when they show persistent surface accumulation rather than the surface depletion observed on ZnO nanomaterials.^23,24^ Of note is that the HoD measurements display a large range of RR with little correlation to the trend of our previous measurements (MoD) that were shown to have *I*–*V* behavior dominated by quantum mechanical tunnelling at the edge of the Au-ZnO interface.^14^ The LoD NWs show a smaller range of RR with a much closer adherence to the previous trend indicating similarity in the transport behavior but with a larger value of R_Au_ ∼ 0.75 at which the contact behavior switches from Ohmic (RR = 1) to rectifying (RR > 1), compared to R_Au_ ∼ 0.6 for the previous MoD NWs. This later onset may likely be explained by a lower concentration of oxygen acceptors on the sloping ZnO facets near the Au interface of the LoD NWs with respect to the MoD NWs due to the lack of O_2_ when cooling to RT from 500 °C making edge tunnelling easier when the LoD Au contact is proportionally larger in diameter with respect to the NW. We previously determined the NW facets surrounding the Au contact to be the sloping facets in the range between {21̅1̅4} and {11̅01} that extend from the NW {011̅0} side facets to join the Au contact interface and (0001) NW top facet.^18^ Facets within this angular range with respect to (0001) have been shown to be highly defective with a high selectivity to oxygen species.^25^ The large range of RR in the HoD NWs indicates that a “one-size-fits-all” transport mechanism is not present with RR influenced strongly by variations in effective carrier concentration, near interface point defects and potential barrier inhomogeneity resulting from defects/donors/acceptors, and surface chemistry influencing the nature of each Au nanocontact depletion region. Our previous work showed with 3D finite-element simulations that a transition from rectifying to low-resistance ohmic transport behavior can be achieved by modulating the surface charge from depletion with charge density of 10^12^ cm^–2^ to a donor-type accumulation of density 10^13^ cm^–2^ on the ZnO facets surrounding the Au nanocontacts.^14^ The comprehensive simulations were able to capture all major conduction mechanisms, including the effect of donor- or acceptor-type defects within the bandgap. The effect of defects alone was not able to replicate the transition from ohmic to Schottky-like behavior without the influence of surface charge. Additionally, an obvious explanation for the wide range of *I*–*V* characteristics could be variations in NW structure and Au-ZnO interface structure and chemistry that would determine the nature of the potential barrier.^26^ The experimental setup is considered to be a M-S-M device structure typically used for single NW piezotronic sensing devices but also free from any NW-substrate interaction that can influence the NW surface potential.^3^ It can be expected that the metal-on-metal contact surface between the STM tip and Au particle is not perfectly intimate with some nanoscale/atomic roughness. Charge transport is dominated by the Au-NW Schottky contact particularly in low forward-bias and reverse-bias, and this is combined with the additional NW resistance that can be in the region of 100 kΩ to MΩs for very thin NWs (<50 nm diameter),^27^ and the ohmic contact resistance at the NW-grid junction.^17^ We can see that the series resistance of these features will be much greater than the STM tip-Au metal–metal contact resistance and can be safely ignored in the analysis. Previous measurements of similar NWs in this configuration showed the Au contacts were stable above ±5 V and >20 μA in forward-bias and only showing features of interface breakdown in reverse-bias that was exhibited as the formation of atomic step-edge defects at the edge of the Au-ZnO interface.^18^ Previous analysis of similar interfaces in detail with atomic resolution aberration-corrected HAADF before and after extensive electrical measurements showing them to be abrupt with no presence of Au impurity atoms in the ZnO and no NW crystalline defects near the interface (point defects are not apparent with HAADF unless the sample is atomically thin or the defect is an atom with a high atomic number in comparison to the surrounding matrix).^18^ This makes it important to correlate imaging analysis with techniques that can identify the presence of impurities, dopants, and point defects with specific energetic emissions, such as CL spectroscopy.

### 3.2 Electron Microscopy and Spectroscopy of Au-ZnO Nanowire Interfaces

The Au particles are frequently composed of two grains once cooled to RT after growth with one grain epitaxially aligned with the NW which presents a twinning boundary that intersects the Au-ZnO interface. This variation in the Au structure frequently presents as a single step edge defect where the twin boundary meets the ZnO interface and could have an effect on the potential barrier such as might be expected from barrier inhomogeneity.^18,26,28^ We studied NWs from the HoDP and LoDP samples after plasma treatment and the final electrical measurements with TEM BF imaging to rule out the presence and influence of major crystalline defects. Figure S3 in the Supporting Information shows two NWs from both samples which display a distinctly different change in *I*–*V* characteristics after plasma treatment. Figure S3a shows a LoD NW that initially has RR = 2.5 and Figure S3b shows an HoD NW with RR = 1.7 that increase after plasma treatment to RR = 4.8 and RR = 220, respectively. The NWs appear quite similar in the images with no discerning features that might explain this difference, and both NWs show abrupt contrast changes in the Au particle that is consistent with the appearance of twin boundaries in BF images. This evidence appears to exclude the twinning feature as the main influence on the changes in the *I*–*V* measurements. However, there still remains the question over the ZnO polarity of vapor phase NWs which is still open to some degree with Sallet et al. reporting Zn-polar NWs grown by MOCVD without Au catalysts and O-polar NWs when a catalyst was employed.^29^ That work appears to be something of an exception when the wide variety of ZnO NW growth methods on various substrates predominantly produce Zn-polar NWs, particularly from vapor-deposition methods.^30^ The necessity to assess the polarity of the ZnO crystal remains of vital importance when forming Schottky contacts especially when it was highlighted that O-polar and Zn-polar nanorods grown by chemical bath deposition (CBD) have different growth point defects because of the higher reaction rate of the Zn-polar surface.^31,32^ The spontaneous nucleation of the metal-catalyzed vapor–liquid–solid method employed here could lead to a mixture of O-polar and Zn-polar NWs and produce distinctly different *I*–*V* behavior and reactions to the plasma treatment. Therefore, the polarity of the NWs was analyzed with high-angle annular dark-field (HAADF) imaging in an aberration-corrected STEM.

**Figure 2:**
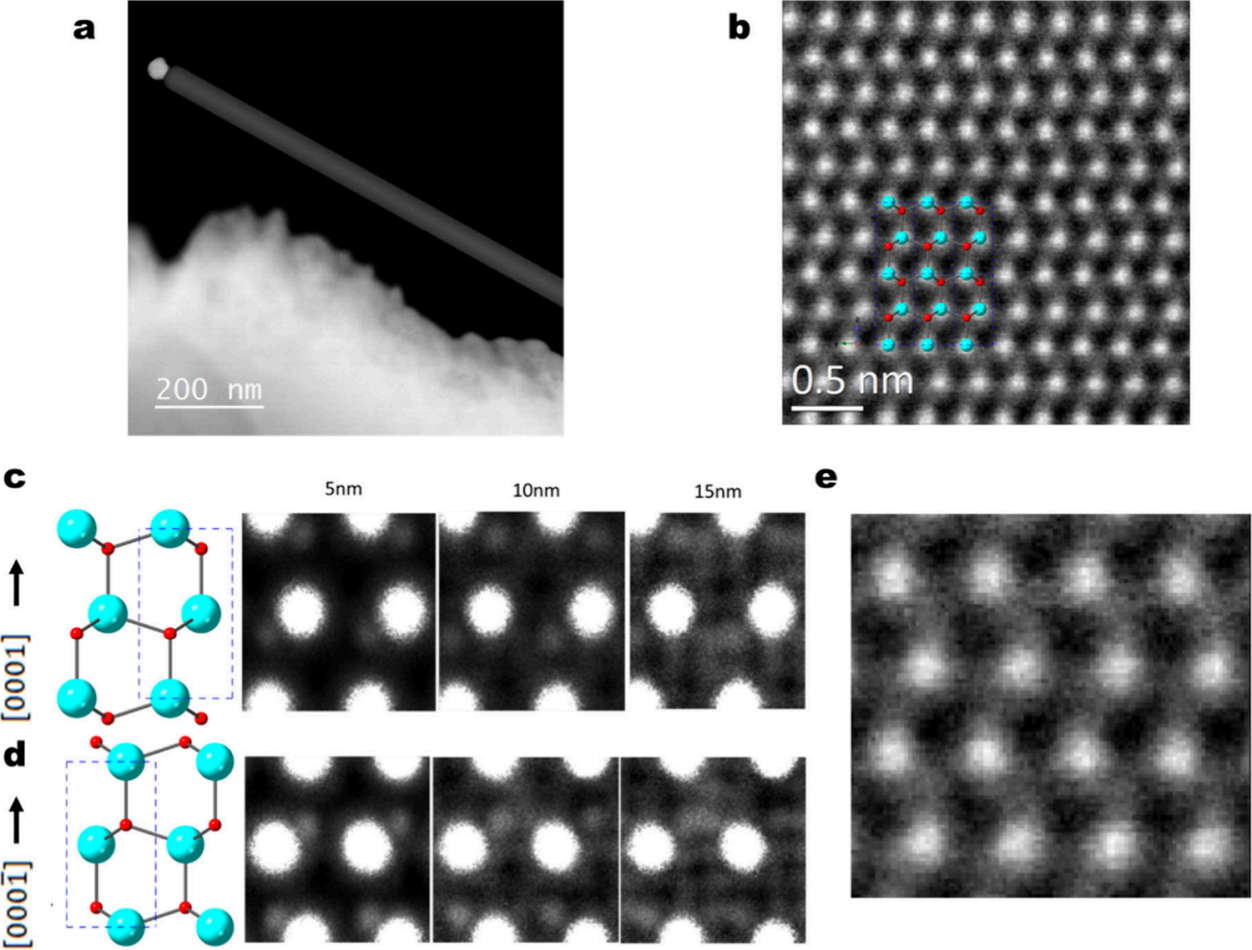
HAADF imaging analysis of a NW located on the [121̅0] zone axis. (a) Low magnification view of the NW with a Au catalyst particle at the growth tip. (b) Atomic-resolution HAADF image of the NW near the edge with a “ball-and-stick” model depicting the atomic arrangement of Zn (blue) and O (red) columns. (c) Simulations of HAADF images of a ZnO slab of various thicknesses on the [121′0] zone axis and [0001]Zn-polar orientation. (d) Simulations repeated for the [0001̅] orientation. (e) Experimental HAADF image at a similar scale to the simulations confirming the [0001]Zn-polar orientation.

HAADF images give contrast dependent on high-angle electron scattering, akin to Rutherford scattering, and their intensity therefore scales approximately as the square of the average atomic number of the imaged material (as ∼ Z^1.7^) such that HAADF is also known as “Z-contrast” imaging. Figure 2a shows a NW orientated on the [121̅0] zone axis, and Figure 2b shows the region close to the Au catalyst particle showing the bright rows of Zn atomic columns. On closer inspection (Figure 2b) an alternating “shadow” below the Zn atomic columns is noticeable, and when we compare this to a ball-and-stick model (Figure 2b) we can see these appear to be O atoms. It is important to validate atomic-resolution images with simulations when interpreting the data “by eye” due to misleading artifacts such as contrast inversion, electron channelling, and noise that may obscure the true nature of the material although these are much reduced for the HAADF technique when compared to BF imaging. We performed simulations using QSTEM^20^ on the same zone axis for various thicknesses representing the maximum estimated ZnO thickness in the analyzed region of this NW, as shown in Figure 2c and 2d. It is apparent that for thinner slabs of ZnO the oxygen columns are easily distinguishable from the Zn columns and appear to validate the experimental images confirming the [0001] growth orientation of this NW pointing toward the catalyst particle. For thicker slabs, channeling effects distort the weaker signal from the low mass of the O atoms such that the position relative to the Zn atoms becomes less clear. The clarity of the O columns is also dependent on the zone axis on which the crystal is analyzed and is displayed by our analysis of a NW on the [011̅0] zone axis, shown in Supporting Figure S4. The simulations of the HAADF imaging on the [011̅0] zone axis show that for thicknesses up to 25 nm the oxygen atoms can still be identified. The HAADF images shown in comparison with the simulations again confirm the Zn-polar orientation. HAADF analysis was conducted on a further three NWs that showed Zn-polar orientation. The TEM and HAADF analyses show there are no major variations in crystal structure, diffusion of Au atoms, or interfacial layers that may account for the large range of RR found in the NWs and crucially in those that do not increase in RR after plasma treatment. This large range in RR is more noticeable for the HoDP NWs with a large fraction remaining Ohmic-like, indicating the additional oxygen annealing step of the LoDP NWs has a considerable effect on the Schottky properties. After ruling out variations in crystal structure and major defects near or at the Au interface we can speculate the large variety in *I*–*V* behavior may result from varying point defects, surface chemistry or individual donors/acceptors near the interface that can have an overriding influence on nanoscale contacts.^33^ However, it is not clear what the dominant donors are in these n-type NWs and whether these are related to oxygen vacancies (V_O_).^6,31^ Therefore, we performed 5K CL on a large number of NWs.

### 3.3 5K Cathodoluminescence Spectroscopy of ZnO NWs at the Au Interface

NWs from both LoD and HoD growth methods were analyzed with 5K CL before and after plasma treatment in the top-down configuration such that the electron beam was focused through the Au particle and near to the Au-ZnO interface. We estimated the depth at which the excitation occurs to be less than 220 nm below the interface from Monte Carlo simulations performed with the CASINO software. Similar analysis of the penetration depth was performed by Cox et al. for CL at 5 kV beam energy focused on a ZnO plane but they also concluded that the penetration depth is significantly reduced when focused through metal overlayers such as the Au contacts here resulting in the maximum excitation occurring closer to the contact interface.^11^ Our work is the first exhaustive set of experimental data correlating the *I*–*V* characteristics of individual NW top contacts below 50 nm diameter with high-resolution optical spectroscopy of the NW material close to the metal-NW interface.

**Figure 3:**
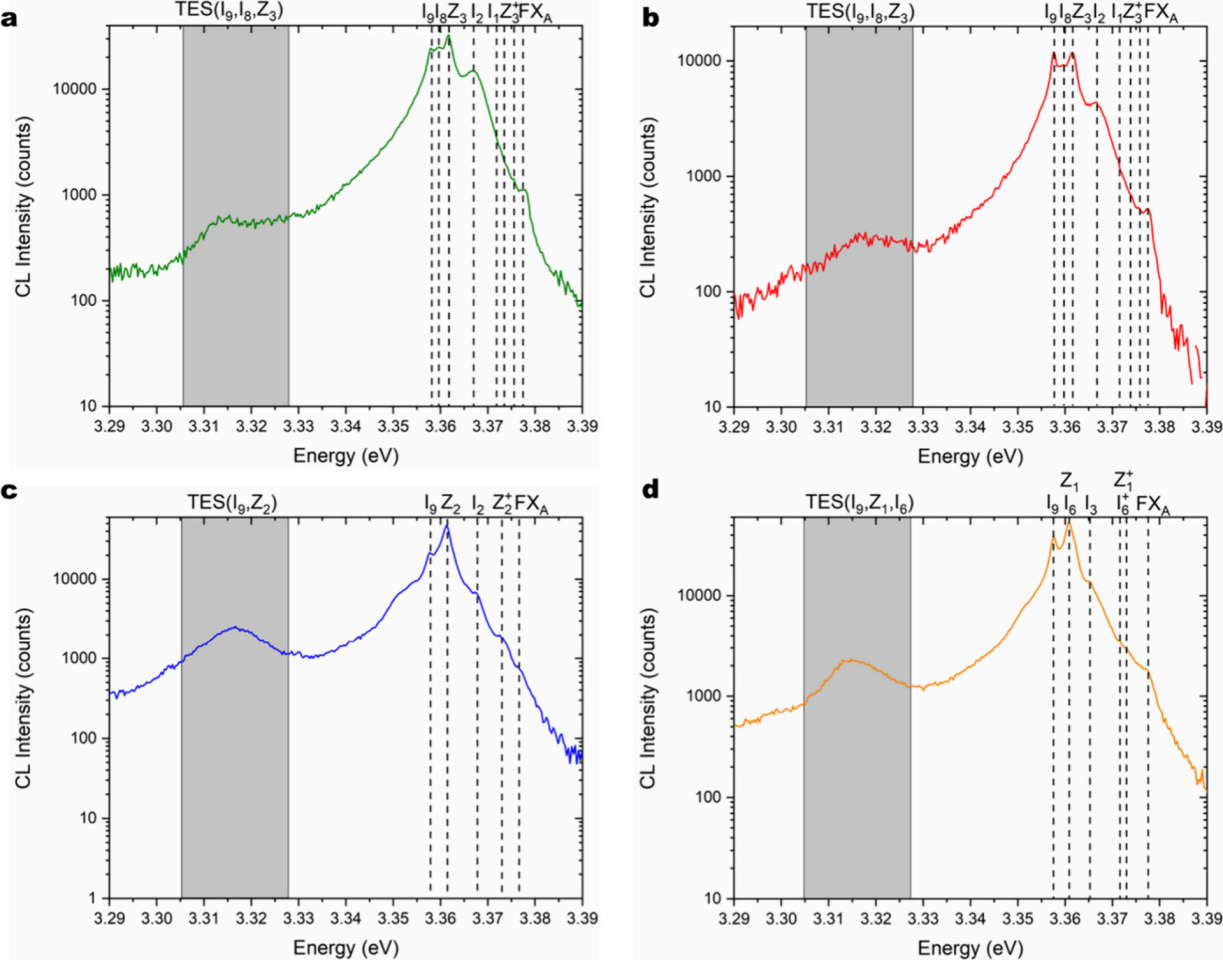
High-resolution 5K CL spectra of the near-band-edge region of individual ZnO NWs in the top-down configuration and focused near the Au-ZnO interface for each sample: (a) HoD; (b) LoD; (c) HoDP; (d) LoDP.

Figure 3 shows the 5K CL spectra for each sample before and after plasma treatment, focusing on the near-band-edge (NBE) emission dominated by radiative transitions of neutral- and ionized-donor bound A-excitons around 3.36 eV.^34,35^ Comparing the HoD and LoD samples in Figure 3a and 3b, respectively, the 5K CL spectra look very similar. The NBE emission is very intense on both samples, and no significant visible emission band is detected (see Figure S5 in the Supporting Information for the full energy range CL spectra including lower energies), which confirms the high crystalline quality of the NWs. The visible emission band is most frequently attributed to point defects in the bulk of the NWs and is observed in other growth methods that typically produce thicker nanostructures where defects can have an overriding effect on electrical contacts, such as those grown by chemical bath deposition and pulsed laser deposition.^11,31^ A statistical analysis by Cox et al. showed that the concentration of point defects leading to radiative transitions in the visible emission band increases with the diameter of ZnO nanorods, which is in very good agreement with the undetectable emissions at energies below the NBE in the thin NWs analyzed here.^11^ This is also corroborated by CL measurements previously made on ZnO NWs grown at high temperature (MOCVD).^36^

The high intensity and high-resolution of the NBE emission allows precise assignment of the excitonic lines associated with residual impurities in the NWs, using the *I* nomenclature.^37^ In both sets of LoD and HoD NWs, radiative transitions involving longitudinal and transversal free A-excitons (FX_A_) are located at 3.377 and 3.375 eV, respectively.^34^ Several excitonic lines are further related to residual impurities involving In (*I*_9_ and *I*_2_ at 3.3577 and 3.3669 eV, respectively) and Ga (*I*_8_ and *I*_1_ at 3.3596 and 3.3715 eV, respectively).^34,35^ The presence of In_Zn_ and Ga_Zn_ dopants is very typical in ZnO,^37^ and likely due to residual contamination in the growth reactor involving previous use of ITO and GaN substrates, respectively. Furthermore, and most importantly, a prominent excitonic line at 3.3617 eV and its associated counterpart at 3.3737 eV can be attributed to carbon species through the respective *Z*_3_ and *Z*_3_^*+*^ lines.^38^ The incorporation of carbon species into NWs might be expected from the carbothermal growth process but is frequently ignored. The introduction of C^+^ into the ZnO NW matrix has been shown to induce magnetic properties that may introduce some intriguing applications for these NWs.^39^ STEM-EELS compositional mapping of NWs that have been exposed to atmosphere reveals a surface layer of carbon species (Supporting Information Figure S6) indicating that the *I*–*V* measurements of the as-produced HoD and LoD samples contained a contribution from increased carbon most likely in the form of chemical interactions with the surface adsorbates and carbon present during the growth process in the form of CO/CO_2_. There is also the somewhat unexpected finding of carbon within the bulk revealed by the CL spectroscopy.

In addition, it is likely that the excitonic line related to residual impurities involving Al_Zn_ dopants (*I*_6_ and *I*_6_^*+*^ at 3.3608 and 3.3727 eV, respectively) occurs in the asymmetrical shoulder of the *Z*_3_ line since the energetic position of both lines is very close.^38,40^ The migration of Al residual impurities from the Al_2_O_3_ crystal substrates may favor the incorporation of Al_Zn_ dopants into ZnO NWs. Correlatively, radiative transitions involving two-electron satellites (TES) related to the *I*_9_, *I*_8_, *I*_6_ and *Z*_3_ lines are located in the range of 3.305–3.325 eV.^34,35,38^ It should further be noted that radiative transitions involving surface excitons (SX) are expected to be around 3.366 eV in the broad band involving the *I*_2_ line. Vacancy defects, specifically V_O_, are known to concentrate at the surface of nanorods^11,41,42^ and it is possible that SX is related to these defects as SX becomes more dominant when the size of the nanostructures is reduced.^43,44^ Although there is growing evidence that V_O_ in the bulk of ZnO may radiate at an energy below the range of CL and provide centers that contribute to nonradiative decay,^45^ radiative transitions involving SX related to V_O_ are particular recombination processes that may emit around 3.366 eV. The as-grown, HoD and LoD samples, hence show very similar spectral features with no distinct differences. The consistency of the CL spectra that show very little variation in emissions across the measured 5 K CL of ZnO NWs is in agreement with the HAADF analysis, revealing the unipolar growth direction and distinct low concentration of point defects.

The oxygen plasma treatment of ZnO NWs clearly results in a very significant increase in the NBE intensity for both HoDP and LoDP samples, as presented in Figure 3c and 3d. This is an indication that nonradiative recombination processes are strongly reduced following the oxygen plasma treatment. More interestingly, the nature of the radiative transitions in the NBE emission region is affected by the oxygen plasma treatment, and the HoDP and LoDP NWs do not exhibit the same behavior. In both cases, a redistribution of the carbon-related defects occurs in the bulk of the ZnO NWs near the Au contact interface. For the HoDP sample, the *Z*_3_ and *Z*_3_^*+*^ lines appear to be replaced by the *Z*_2_ and *Z*_2_^*+*^ lines at 3.3612 and 3.3731 eV, respectively.^38^ For the LoDP sample, the *Z*_3_ and *Z*_3_^*+*^ lines appear to be replaced by the *Z*_1_ and *Z*_1_^*+*^ lines at 3.3608 and 3.3731 eV, respectively.^38^ Interestingly, following the oxygen plasma treatment, the broad band involving the *I*_2_ and SX lines is much less pronounced. The presence of the *I*_3_ line at 3.3651 eV in the LoDP sample is even noticed. The intensity of SX (I_SX_) when compared to the intensity of the dominant line in the NBE (I_NBE_) is of noticeable interest when the four samples. Comparing the absolute intensity of the NBE emission to the SX emission (I_NBE_/I_SX_) we can see from Figure 3 that the HoD sample is 32764/14907 = 2.2, LoD sample 11862/4422 = 2.7, HoDP sample 47325/6292 = 7.5, and LoDP sample 53203/13708 = 3.9. The higher I_NBE_ and I_SX_ of the HoD sample can be explained by more intense Z lines due to increased carbon and increased V_O_ (both shown later by XPS) and a smaller intensity ratio. The lower I_NBE_ of the LoD sample has lower intensity Z lines and fewer V_O_, with less carbon and V_O_ due to the annealing treatment also shown later by XPS. The effect of the oxygen plasma treatment is very clear on the HoDP and LoDP samples with respect to the HoD and LoD samples, by boosting the I_NBE_/I_SX_ ratio. The difference between the HoDP and LoDP samples is less straightforward (i.e., 7.5 vs 3.9) with additional and less well understood contributions also playing a role. This may indicate that the oxygen plasma treatment improves the stoichiometry by filling V_O_ throughout the NWs, both in the bulk and near the surface. The fact that both HoD and LoD samples generally exhibit a significant increase in rectifying behavior after oxygen plasma treatment is unsurprising since it predominantly acts on the surface (especially when the NWs originally had high crystalline quality), which is confirmed by the small differences in the CL data (i.e., nature of the *Z* lines). Therefore, it seems likely that the range and scatter of RR values, particularly in the HoD NWs, occurs due to the interplay of low concentrations of point defects near the interface and surface effects surrounding the contacts, particularly the ZnO facets near the Au contact interface.

To confirm the CL analysis, the NBE emissions were compared to 4 K photoluminescence (PL) spectroscopy of the MoD sample that was cooled to RT in the presence of oxygen at the end of the growth process, shown in Supporting Information Figure S7. The PL and CL data are in very good agreement. In particular, the *I*_9_ line assigned to In_Zn_ is confirmed with both techniques while the CL data further confirms this with the *I*_2_ line. The *I*_6_ line can be assigned to Al_Zn_ and occurs in the asymmetrical shoulder of the *Z*_3_ line with both lines very close in the energetic position. The broadening of the TES line toward higher energy, particularly in the LoDP NWs, supports this argument. It should also be noted that the PL data also collects emissions from the ZnO base layer growth that covers the substrate and is highly likely to contain Al impurities migrating from the Al_2_O_3_ crystal during the high temperature growth, however, these impurities were also detected in the 5 K CL emissions from the NWs close to the Au contact interface.^46^ The SX line that can also be seen in the PL spectra is recombination due to surface bound excitons and occurs at the same energy as the *I*_3_ peak.^37^ The energy of the SX emission at 3.366 eV is too high to be associated with H_O_(*I*_4_) or V_Zn_-H_n_ and also H_BC_ at 3.360 eV that are common donors in CBD grown nanorods.^47^ The concentration of V_O_ at the surface would appear to be crucial to understanding the effect of oxygen annealing and plasma treatment on the electrical transport properties of these nanocontacts. Annealing in O_2_ is expected to improve the overall stoichiometry filling V_O_ throughout the NWs, both in the bulk and near the surface, while the plasma for relatively short treatments has a predominantly surface effect.

Therefore, with the bulk/near interface donors assigned predominantly to In_Zn_ and Ga_Zn_ impurities, the plasma can have several effects, one of which is initially removing loosely bound/chemisorbed species and then roughening the surface when subjected to increased processing time. Longer plasma treatments are expected to lead to a higher concentration of bonding sites that can accommodate OH reducing any surface depletion and producing highly conductive NWs with little or no current rectification.^48^ The optimized oxygen plasma treatment applied here was shown not to have any roughening effect on the NW surface (see Supporting Information Figure S8 showing an atomic-resolution bright-field STEM image of an LoDP NW and the atomic regularity at the surface and very low roughness). Filling V_O_ also occurs but this does not directly affect resistivity as V_O_ are deep donors in ZnO NWs but removing these vacancies near the surface improves the surface stoichiometry and reduces the variability in available bonding sites.^6^ Donor impurities can exist by substituting O or Zn and interstitially in which case displacing donors at V_O_ sites may to some degree increase resistivity while donors at Zn sites and interstitially will remain untouched helping somewhat to explain the inability of the oxygen plasma to increase resistivity in these NWs.^49^ This is shown by estimating the resistivity from the current at +1 V for each NW M-S-M structure. The HoD sample had an average resistivity of 3 Ωcm and the LoD sample of 9 Ωcm, and both decreased after plasma treatment to 1.8 and 6.3 Ωcm, respectively, even though current rectification increases substantially revealing that carrier concentration is not the main contributing factor to the quality of the Au rectifying contacts. This is a crucial fact when quantum mechanical tunneling is dependent on carrier concentration.

CBD grown nanorods have donor-type substitutional hydrogen on oxygen sites (Ho) that are annihilated at the surface to some degree by oxygen plasma and it is easy to see that the resistivity increases when these vacancies are filled by energetic oxygen ions.^13,31^ CBD nanorods also exhibit a high proportion of hydrogen at body-centered lattice sites or depending on growth polarity hydrogen can be complexed to V_Zn_. Lavrov et al. have shown that H_BC_ can show a photoluminescence at 3.360 eV while the H_O_ emission at 3.362 eV (I_4_ line) is not apparent in the CL or PL spectra of the NWs analyzed here.^47^ The oxygen plasma may increase the concentration of V_Zn_ but with no obvious source of donor impurities after the growth process when the NWs have been removed from the substrate, it would appear any Zn vacancy defects cannot be filled with additional donors. Therefore, donors occurring after/during plasma treatment most likely come from OH on the surface, and it is to be expected that the additional annealing step applied to the LoD NWs will fill many V_O_ in the bulk and at the surface greatly reducing the scatter and range of RR. These conclusions are indirectly confirmed when comparing the small differences in the CL spectra that result from the plasma procedure.

The reduction in resistivity differs to our previous work on CBD grown nanorods that are dominated by hydrogen donors related to vacancy defects and interstitial donors^50^ which increased in resistivity after a similar plasma treatment, but similar to the NWs here they also showed a considerable improvement in Schottky contact behavior.^31^ In those nanorods, the Schottky contact behavior was influenced by the near-interface defects and variations in donors according to the nanorod crystal polarity. However, in the NWs here that have a much smaller diameter, different growth method, and epitaxial alignment with the Au interface, the surface of the NW will have a much greater influence and was analyzed with X-ray photoelectron spectroscopy (XPS).

### 3.4 X-ray Photoelectron Spectroscopy of ZnO Nanowire Arrays

The HoD and LoD samples were analyzed with XPS in the as-grown state before further analysis was conducted immediately after they were subjected to the plasma treatment. XPS scans were then performed on the HoDP and LoDP NWs at 12 h intervals after loading over a period of 48 h to investigate any transient effects. In this section, we report the results of the O 1s, Zn 2p3/2, and C 1s peak analysis for the HoD and LoD samples together with the HoDP and LoDP zero-hour scans (immediately after loading). The zero-hour HoDP and LoDP data was chosen as the XPS chamber conditions (10^–9^ mbar) most closely represent the surface chemistry of the NWs in the UHV multiprobe instrument (10^–11^ mbar) used for the electrical measurements. The results of the peak analysis are summarized in Table 1.

**Table 1:** Data Summarizing the XPS Peak Analysis for the Four Sample Types^a^

Sample	HoD	HoDP	LoD	LoDP
O_Zn_ (Δ) (eV)	530.56 (−)	530.53 (0.03)	530.57 (−0.01)	530.49 (0.07)
% O_S1_	51.1	47.5	44.8	40.6
Zn 2p3/2 (Δ) (eV)	1021.48(−)	1021.44 (0.04)	1021.49 (−0.01)	1021.37 (0.11)
C 1s (Δ) (eV)	285.48 (−)	285.13 (0.35)	285.51 (−0.03)	285.04 (0.44)
C:(Zn+O)	0.59	0.11	0.53	0.12
(O_S_+O_Zn_):Zn	1.43	1.22	1.37	1.19

a Shifts are referenced to the as-grown HoD sample. Data for the HoDP and LoDP are from the zero-hour XPS analysis immediately after plasma treatment and achieving 10^–9^ mbar chamber pressure.

Table 1 shows that the surface sensitivity of XPS reveals a considerable change in the surface related oxygen (O_S1_), the concentration and peak position of carbon (C 1s), and ratio of total oxygen to zinc for both the oxygen annealing and plasma treatments when compared to the HoD sample. In particular, we can see from the ratio of total oxygen to zinc (O_S_+O_Zn_):Zn that an improvement in surface stoichiometry is observed with both the annealing and the oxygen plasma procedures. This can be explained by analyzing the O 1s and C 1s peaks more closely.

First, and most importantly to aid the interpretation of the O 1s components, the most interesting aspect of the C 1s peak data are the plasma treated samples. After plasma treatment of both the HoD and LoD NWs the analysis shows a large reduction of total carbon compared to the total combined Zn and O from a ratio >0.5 to nearly 0.1 (at ∼0 h after plasma treatment). Within the XPS chamber, the C 1s peak increases from ∼10% of the combined C 1s, O 1s, and Zn 2p/3 peaks immediately after plasma treatment to ∼25% after 48 h. This corresponds with an increase of the higher BE shoulder of the O 1s peak associated with surface carbon species and H_2_O (O_S2_) from ∼1% of total O to ∼3–4% 48 h later. After 24 h in the XPS chamber C 1s had risen to 20%. The XPS chamber has a pressure of 10^–9^ mbar, a higher pressure than the UHV chamber, and carbon will grow on the surface at a much greater rate in the XPS. The C 1s peak shifts immediately after the plasma treatment to ∼0.35 eV lower BE for the HoDP sample and ∼0.44 eV for the LoDP sample due to the removal of loosely bound carbon molecules and chemisorbed carbon. The BE shift indicates a chemical change in the carbon layer and a slight variation of the HoDP and LoDP sample surfaces possibly due to the difference in bonding sites that the annealing creates. This shift is then counteracted over 48 h by the growing carbon layer with each of the core-level peaks shifting by ∼0.1 eV to a higher BE as the carbon layer develops.

**Figure 4:**
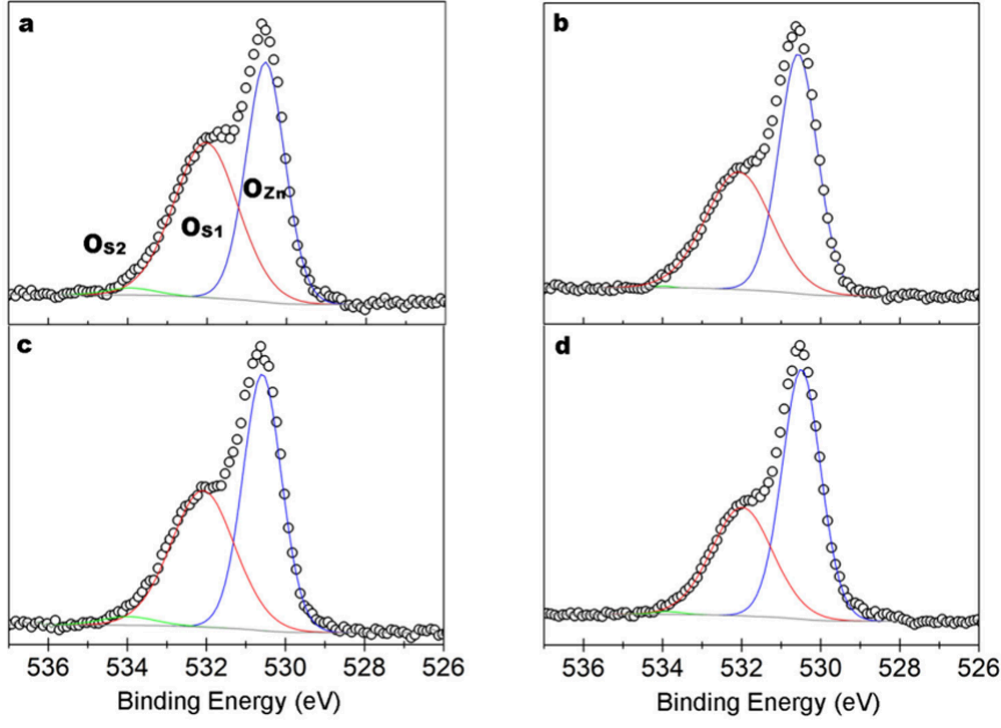
XPS data (black circles) of the O 1s peak including the Gaussian peaks fit to match the envelope to the experimental data comprising of O_Zn_ (blue), O_S1_ (red) and O_S2_ (green) before (a,b) and after (c,d) oxygen plasma treatment of the HoD and LoD samples, respectively.

The O 1s peak was fitted with three components: the main O 1s peak associated with lattice oxygen (O_Zn_), the surface oxygen peak associated with oxygen ions and hydroxyl bonds (O_S1_), and the higher binding energy peak associated with surface adsorbates such as carbon molecules and water (O_S2_), shown in Figure 4 for each sample.

The total O compared to Zn decreases with the additional oxygen annealing step of the LoD sample, and both LoDP and HoDP samples exhibit a larger decrease in total O after plasma treatment (see (O_S_+O_Zn_):Zn in Table 1). The raw data of the Zn 2p3/2 peaks comparing both samples before and after plasma treatment are shown in Supporting Information Figure S9 which displays the increased emissions at this energy and the small shift in peak energy after plasma treatment. The proportion of oxygen O_S1_ is often used to determine chemical changes to the ZnO surface, and in the as-grown HoD sample, it is 51.1% of total O while for the LoD sample, it is 44.8% of total O. The plasma treatment decreases this further to 47.5% and 40.6% for the HoDP and LoDP NWs, respectively. This coincides with the elimination of O_S2_ and the near-complete removal of carbon. The reduction of O_S1_ relative to O_Zn_ indicates a removal of surface oxygen species such as hydroxyls while O_S1_ is also associated with O^2–^ ions at oxygen-deficient sites and therefore V_O_ that decreases with annealing and then further with plasma treatement.^51−53^ Filling of V_O_ and the decrease in O_S1_ leads to a more balanced surface stoichiometry that is apparent in the change of (O_S_+O_Zn_):Zn which decreases with both annealing and plasma treatment, consistent with O_2_ based cleaning methods of ZnO surfaces typically used to achieve rectifying contacts.^22^ The overall reduction in oxygen is most likely due to the cleaning effect removing oxides of carbon, water, and other weakly adsorbed species while filling oxygen vacancies that contribute to the O_S1_ peak. The native contamination layer on ZnO is known to be predominantly adventitious carbon and hydroxyls with hydrogen bonding to surface oxygen ions (H+O^2–^ → OH^–^ + e^–^) donating electrons and increasing the carrier concentration in the surface space charge region.^22^ In opposition to this, oxygen also adsorbs as O^2–^ and O^–^ at defect sites acting to create the depletion layer found on ZnO NWs.^22^ The reduction of the O_S1_ peak with annealing and subsequent plasma treatment appears to be consistent with the reduction of V_O_ found in experiments of oxygen-annealed ZnO were it has been related to Zn–O bonds neighboring V_O_.^10,54^ V_O_ can in some cases contribute to n-type doping particularly in the case of substitutional hydrogen; however, these emissions were not apparent in the CL spectra. Compared to the annealing treatment, the oxygen plasma induces the greatest reduction in near surface V_O_ that can be expected to decrease the positive space charge near the surface and alter the Fermi level pinning caused by subsurface vacancies and the adsorption of atmospheric species. Additionally, a reduction in dangling bonds and thus reaction sites for adsorbed species results in a decrease in NW resistivity, which also indicates there is no action on the dominant bulk donors.

Annealing is expected to improve the bulk crystal quality, and on exposure to air, hydroxyls will still form and surface defects will also bond with oxygen; however, the much lower concentration of O_S1_ of the LoDP NWs suggests a much lower density of bonding sites. The exact atomic nature of the surface will determine the resulting balance of acceptors and donors, and determine whether the surface is depleted or accumulated.^23^ Therefore, the surface potential is expected to vary from NW-to-NW but more so between each of the samples, which the XPS analysis captures as an average sampling of many NWs. Annealing in O_2_ may in addition to filling bulk vacancy defects react with the remaining surface defects and dangling bonds. A number of works reveal the localization of V_O_ near the ZnO surface^41,55,56^ and show that the XPS O_S1_ peak is a V_O_ emission that correlates well with the data here showing a decrease of O_S1_ after annealing and plasma treatment.^10,48^

### 3.5 Discussion and Analysis of the Experimental Results

To assess the quality of the Schottky contacts the standard terms of the effective potential barrier height (ϕ_e_) and ideality factor (*n*) were calculated for each contact by fitting standard thermionic emission theory to the current–voltage data for each measurement in the forward-bias regime where the *I*–*V* characteristics show an exponential increase.^57^ It should be noted that the experimental configuration here is effectively a M-S-M device configuration commonly used for single NW piezotronic and piezo-phototronic sensor devices with one Schottky barrier at the Au-ZnO interface in series with the NW and ohmic NW-to-grid contact.^3^ This results in the applied voltage being the sum of the voltage drop across each of the components as follows:
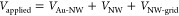
2

Therefore, the effective potential barrier height reported and analyzed here is that of the whole M-S-M structure (eq 2) and not strictly the Schottky barrier height of the Au-NW interface as it is not possible to determine the exact series resistance (R_S_) of the individual NWs and contacts for each measurement in such an experimental configuration. It is possible to estimate R_S_ from the *I*–*V* data at higher forward biases where the forward bias potential exceeds the Schottky barrier height, however, this was not possible to perform accurately here as the range of voltages used were maintained at ±1 V and in some cases the *I*–*V* data can still be seen to be diverging from the exponential relationship. This bias range was chosen to ensure resistive heating of the M-S-M structures was minimized and any point defect migration was limited such that transient effects on the transport properties could be monitored with confidence.^11,18^ In cases where the series resistance is non-negligible we can use eq 3 that modifies the standard thermionic emission theory such that the effective barrier height (ϕ_e_) and ideality factor (*n*) can be calculated as follows:
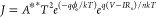
3typically fit to the experimental data in the range  where T is the temperature, q is the electron charge, k is the Boltzmann constant, A** is the modified Richardson constant, however, it is acceptable to use the theoretical value A**=32 A cm^–2^ K^–2^. Examining the example *I*–*V* data in Supporting Information Figure S2 of some NWs, particularly after plasma treatment where the M-S-M structures are highly rectifying, the *I*–*V* data below 0.5 V are unreliable as the noise level of the system is ∼20 pA. Such low measured current is a consequence of some NW structures having such small diameters. The data does show that the exponential relationship is held at higher forward-bias voltages such that the exponential relationship is evident which is used to calculate the effective potential barrier and ideality factor of the entire M-S-M structure. The *I*–*V* measurements show the exponential relationship in forward-bias does not hold up to the +1 V maximum applied potential indicating the flow of charge is then unrestricted by the potential barrier at those applied voltages. However, with respect to enhancing useful devices it is not necessary to know the exact Schottky barrier height at the Au nanocontact to NW interface while applying the techniques investigated here to improve rectification by several orders of magnitude and increase the effective potential barrier height of the devices. In fact, when we consider the effect surface charge can have on the contact depletion region it is very difficult to knowingly determine the exact effective Schottky barrier height of the Au nanocontact to NW interface unlike much larger contacts to nanorods and planar single crystals.^14,58^ To create an accurate theoretical model of the potential fields and electrical transport in Au-NW contacts/M-S-M structures it would be important to determine the exact nature of the Au-NW depletion region particularly from the effective NW carrier concentration and surface depletion such that any edge-effect potential barrier thinning and edge-tunnelling can be taken into consideration.^14^

**Figure 5:**
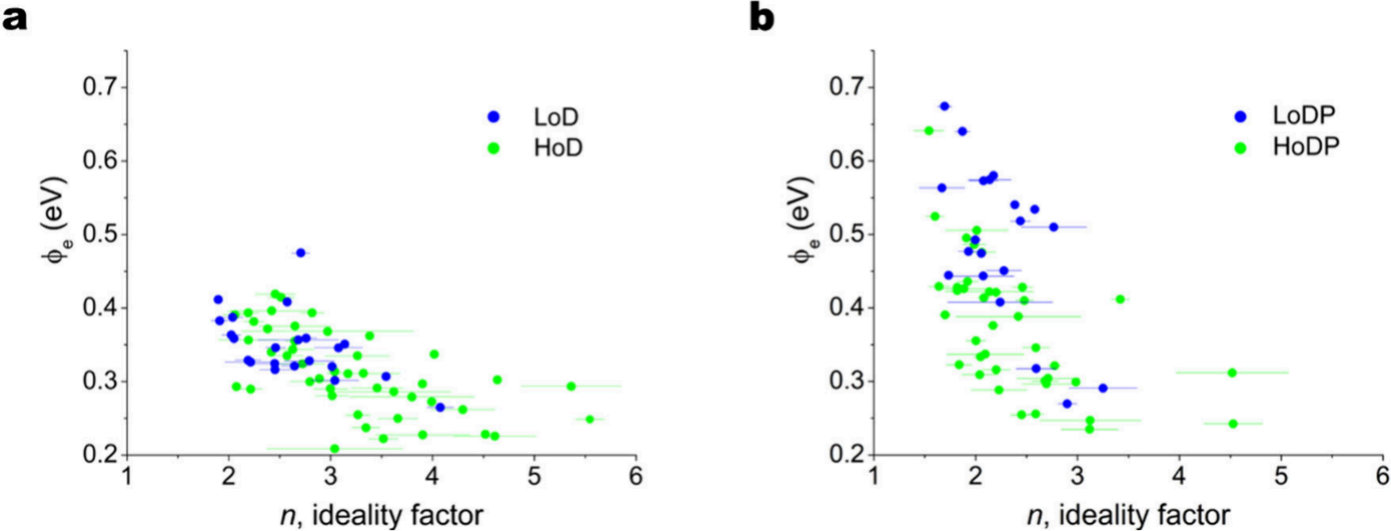
Plots of the increase of ϕ_e_ with *n*, (a) before plasma treatment and (b) after plasma treatment for the two NW types showing a large increase in ϕ_e_ and reduction in *n* for the majority of the NWs after the optimized plasma treatment. The error bars for *n* indicate the standard deviations of values from the experimental data.

Before plasma treatment the HoD and LoD NWs had an average ϕ_e_ of 0.32 and 0.35 eV, respectively. Plasma treatment increased ϕ_e_ for both samples to an average of 0.37 and 0.49 eV. The calculation of ϕ_e_ is sensitive to NW conductivity and is one possible explanation of the initial difference between samples before plasma treatment. However, we see that overall, the resistivity of both HoD and LoD samples decreases after plasma treatment and therefore cannot explain the 0.14 eV average increase of ϕ_e_ in the LoD NWs. The calculated ϕ_e_ for each NW is plotted against *n* in Figure 5 showing a clear increase in ϕ_e_ and improvement in rectifying ideality (*n*) after plasma treatment (Figure 5b). The estimated resistivity values for the HoD and LoD samples are 3.02 and 8.99 Ωcm, respectively, before plasma treatment and 1.75 and 6.34 Ωcm after plasma treatment. Four-probe measurements are free of contact resistance and reveal NW resistivity. NWs below 60 nm diameters have been revealed to have resistivity between 0.1 and 4 Ωcm.^27^ This is in good agreement with the values measured here when we consider the additional resistance of the Au-NW interface and the NW-grid ohmic contact. AFM measurements of vertical ZnO NWs with Au catalyst contacts show the maximum “On” current at +2 V is ∼2 nA which decreased after applying a compressive force to the Au contact with the AFM tip.^59^ The work is an excellent example of the piezoelectric effect acting to modify the Au-NW Schottky contact that we avoid here by approaching the STM tip in the horizontal position with tunneling feedback to detect physical contact between the tip and Au particle. The data shows ϕ_e_ has very little correlation with estimated resistivity and any relationship that may exist is even less apparent after plasma treatment. Similarly, there is no correlation of resistivity with ideality factor that would suggest tunneling through the depletion region at low bias. In addition to this, RR has no relationship with estimated resistivity indicating that carrier concentration and therefore tunnelling through the central contact depletion region are not the major influence on RR or ϕ_e_. The oxygen annealing has increased the resistivity, while plasma treatment acts to reduce resistivity but only by a relatively small amount for the optimized treatment. The XPS data show that both oxygen annealing and oxygen plasma reduce the surface carbon potentially freeing surface sites to bond with different atmospheric species. As expected, ϕ_e_ does increase with RR and this becomes more pronounced after plasma treatment, however, there is no correlation between ϕ_e_ and R_Au_. This is to be expected because it is generally quantum mechanical tunneling (depletion width dependent and/or defect-assisted) through the depletion region, the dominant nonideal transport in highly doped semiconductors, at low bias that affects ϕ_e_. The contribution of tunneling at the contact edge that can significantly reduce RR is dependent on R_Au_ and the nature of the surrounding facets. The voltages applied here do not reach the negative bias that might cause reverse-bias breakdown seen in large scale contacts and that has been shown to occur in Au-ZnO NW nanocontacts at approximately −3 V.^18,57^ It should be noted that the potential barrier heights are the effective potential barrier presented by three resistors in series in the form of Schottky contact/NW/ohmic contact to grid.^3^ To determine the exact properties, of the Au-NW Schottky interfaces would require detailed knowledge of the NW bulk properties, surface potential of the NW side and sloping facets to determine the properties, with simulations, or the experimental measurement of the potential drop across each junction of the M-S-M structures, which is complicated by the size of the NWs particularly the Au nanocatalyst contacts.

The NWs with low ϕ_e_ and low RR are not “Ohmic”, i.e. linear *I*–*V*, and they show an exponential relationship in the low forward-bias regime, but they have weak reverse-bias characteristics with a steadily increasing leakage current. Figure 5a shows a range of ϕ_e_ for all NWs of ∼0.2 eV to ∼0.4 eV before plasma treatment and this range expands considerably after treatment (Figure 5b) up to a maximum of ∼0.65 eV with the greatest improvement in Schottky characteristics seen on the LoDP NWs. The effect of the plasma is similar to that seen on nanoislands when the surface surrounding the contact is made insulating and surface conduction is eliminated, see Supporting Information Figure S2 for example *I*–*V* data.^60^ It is interesting to note that before plasma treatment the ideality factor reaches a minimum of ∼1.9 and after treatment this decreases to ∼1.5 with a greater number of NWs nearing this lower value with *n* showing a distinct relationship with ϕ_e_ and RR. Ideality factor is sensitive to tunnelling at low bias and RR is particularly sensitive to tunnelling in reverse bias which indicates a plasma effect on the ZnO surface near the contact edge reducing the “pinching-in” effect of the contact potential field at the interface edge that can lead to “edge-effect” tunneling.^14,58^ The experimental evidence of decreasing barrier inhomogeneity found on planar contacts can lead to a linear relationship of decreasing *n* and increasing ϕ_e_ based on the principles of Tung’s general theories.^28,61^ The contacts here are not large enough to contain numerous “patches” of low potential barrier that would be revealed in the plots shown in Figure 5 and instead are either single crystal or composed of one twinning boundary. Additionally, ideality factor is determined from the exponential *I*–*V* data at low forward-bias which in nanometric contacts is strongly affected by tunneling at the contact edge, capacitance, and defect energy states within the bandgap near the interface.

**Figure 6:**
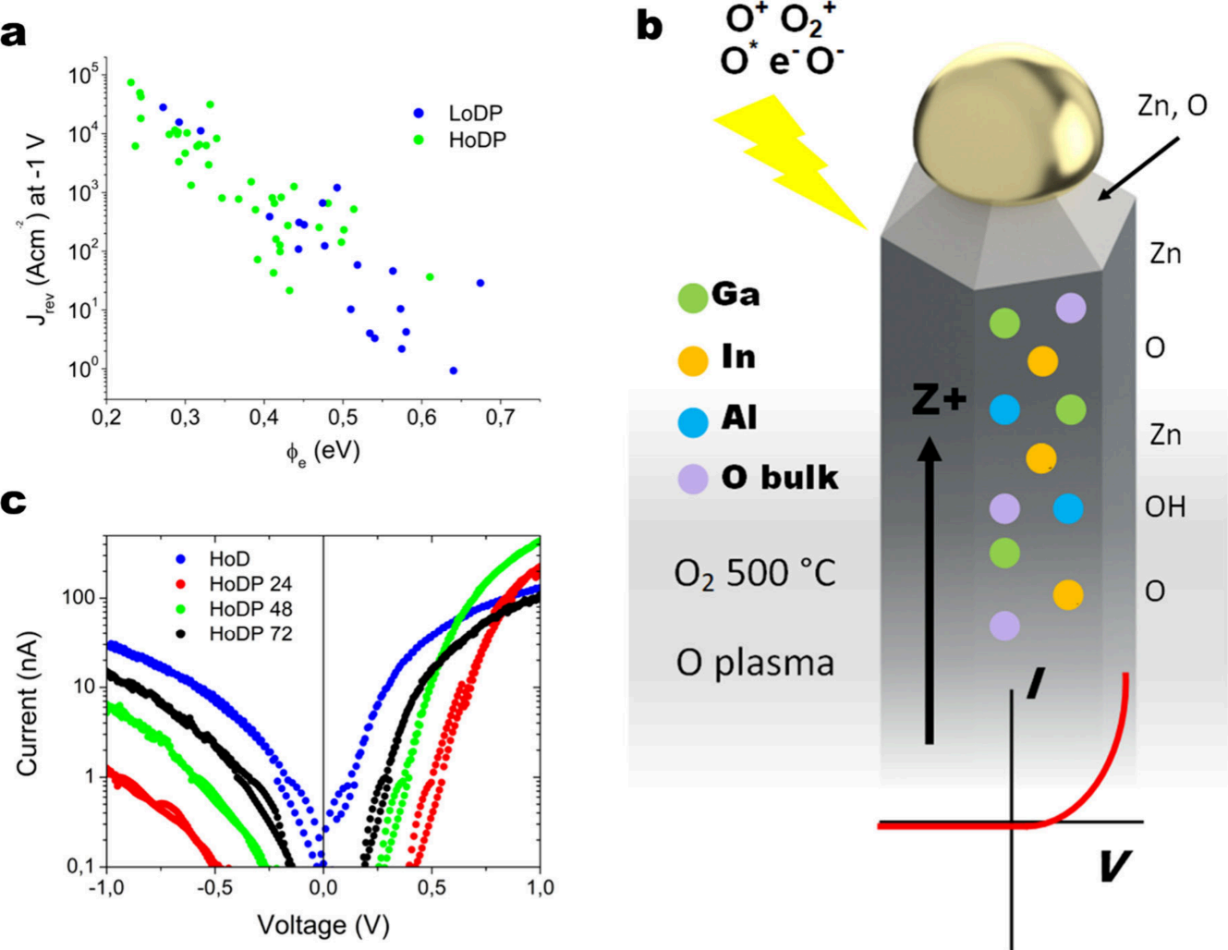
(a) Reverse-bias current density (*J*_rev_) showing a decreasing exponential relationship with increasing effective barrier height ϕ_e_ for the HoDP (green) and LoDP (blue) NWs. (b) Schematic diagram illustrating the major effects on the increased Schottky behavior of the LoDP NWs. (c) Current–voltage plot showing the initial transport behavior (blue) of an HoD NW and the resultant characteristics 24 h (red), 48 h (green), and 72 h (black) after the plasma treatment.

Thermionic emission theory predicts reverse-bias current should saturate in the ideal case at
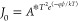
4and Figure 6a shows that the reverse-bias current at −1 V holds an exponential relationship with ϕ_e_ which follows *J*_rev_ ∼ *e*^–23.69ϕ_*e*_^. In the nonideal case reverse-bias current density (*J*_rev_) deviates from eq 4 predominantly due to tunnelling current especially in highly doped semiconductors such as the NWs here with a carrier concentration of ∼10^17^ - 10^18^ cm^–3^ creating a thin depletion region which could be exacerbated by point defects/donors in the space charge region. Although the error in the thermal voltage term is quite large (∼2 times larger) it is remarkably similar to the exponential relationship found on CBD plasma-treated nanorods with deposited Au contacts where the relationship of *J*_rev_ ∼ *e*^–24.12ϕ_*e*_^ was found for O-polar nanorods.^31^ This is quite striking when we consider the nanorods had a diameter in the region of ∼600 nm, while the NWs here have diameters in the range of ∼25–65 nm. With the CBD nanorods and vapor-phase NWs having similar carrier concentrations, it is a good indication that the plasma treatment has nullified any edge effects that would be more apparent in the nanometric contacts measured here.

The size of the contacts here are generally in the range of 500–2500 nm^2^ typically at the lower end of investigations of nanoislands on single crystal wafers that have shown suppressing surface leakage can increase the Schottky barrier height to 0.5 eV and reduces the ideality factor to 2.^60^ These values are similar to those measured here, and the scatter observed in the data is fairly typical for nanocontacts to NWs. However, no overall trend is found in the plasma treated NWs between the effective potential barrier height and contact area or diameter that would indicate electrical transport dominated by a surface effect or variations in dopant spatial distributions that may dominate the *I*–*V* characteristics.^33,60^ Additionally, the contact properties are generally improved significantly after plasma treatment without affecting the resistivity, indicating the dopants in the lattice are unaffected. There is very little relationship between the electrical transport properties and the physical geometric properties of the NWs other than the LoD sample varying with R_Au_. Brillson et al. showed that oxygen plasma treatment of ZnO single crystal before depositing Au contacts was necessary to change the contacts from ohmic to Schotkky-like.^62^ ZnO that was deemed to have high or low defect concentrations depending on the relative CL intensity of visible band emissions was shown to have ϕ_e_ = 0.48 eV and n = 1.3, and ϕ_e_ = 0.43 eV and n = 3.7, respectively. Although visible band CL emissions were not detected in the NWs here, it is possible very low concentrations present after the plasma treatment, being randomly dispersed by nature, may have a substantial effect on the range of ϕ_e_ and *n* for these NWs. The oxygen plasma will reduce the effect of these defects while also acting on the surface defects and chemistry. Various other Au contacts on ZnO provide Schottky contacts with 0.5 eV<ϕ_e_< 0.67 eV and 1.03 < *n* < 1.86 depending on the deposition and surface cleaning methods.^63^ Leonard and Talin investigated Ge NWs with Au catalyst tips with their measurements using a single metal probe and showed ideality factors that increased from 2 to 4 when the NW diameters were reduced from 100 to 30 nm. Ge has the interesting property of displaying large Schottky contacts ϕ = 0.59 eV regardless of the metal due to strong Fermi level pinning close to the valence band. At 30 nm diameter the Ge NWs had *n* ranging between 2 and 4 with rectification of approximately 10–20 at ±0.4 V.^64^ This summary shows the values measured here have properties close to those achievable on singe crystal ZnO, in some cases exceeding them, while improving on the current magnitude and rectification at low voltages for single ZnO NW devices which is desirable for strong signals in various applications.^3^

It has been shown by several studies that the distribution of donor or acceptor-type impurities near to a nanocontact can have an overriding influence on the contact properties resulting in Ohmic, Schottky or blocking type behavior.^8,11,33^ The distribution of In, Ga, and Al near to the Au-ZnO contacts here will undoubtedly impact the measured electrical properties and will likely play some role in the distribution of ϕ_e_, *n*, and RR. There is also the unknown influence of the carbon species present in the bulk revealed by the *Z*_3_ and *Z*_3_^*+*^ lines in the CL analysis^38^ that is frequently ignored and may introduce local electric fields especially when it has been shown that C^+^ in the ZnO NW matrix can induce magnetic properties.^39^ Additionally, the nature of the facets surrounding the Au contact will also play a major role in the electrical behavior with different crystallographic facets that slope from the side {011̅0}to the interface presenting a range of defects, surface steps, and terraces leading to different surface chemistry and thus electrical potential field surrounding the contact.^25,65−68^ We have shown the impact of these facets on the *I*–*V* characteristics in our previous work that examined in atomic detail the same NWs before and after modifying the sloping facets with etching. This negates any possible variation in the distribution of donors/acceptors in the bulk near the interface as the overriding factor on the transport properties in those NWs.^17^ However, the two-step process employed here to reduce bulk V_O_ defects with annealing and surface chemistry modification with oxygen plasma has improved the overall quality of the abrupt epitaxial interfaces to something approaching the quality possible on single crystal large scale contacts, schematically described in Figure 6b that highlights the dominant features acting on the LoDP contacts.^8,63^ Further work is necessary to ensure this improvement is permanent with techniques that may cause surface pinning at a desired energy level and/or passivation such as atomic layer etching (ALE), atomic layer deposition (ALD) or surface functionalization such that the NWs retain their properties in air or other process atmospheres.^69,70^ Examples of the nanometric uniform films that ALD can form on these ZnO NWs are shown in Supporting Information Figure S10.

The requirement for stabilizing Schottky contacts against transient effects was shown by Schultz et al. revealing that PtO_*x*_ on zinc–tin oxide diodes improved their rectification properties with time over several weeks. The aging process could be accelerated with application of a reverse-bias voltage drawing oxygen atoms from the oxidized contact into the depletion region leading to a decrease in carrier concentration near the depletion region.^7^ However, the Au nanocatalyst-ZnO NW contacts have an atomically abrupt interface with no oxide present in the pure Au contact. Measuring the *I*–*V* characteristics of several NWs over a period of 72 h after plasma treatment showed that weakening of the rectifying properties can occur such that after 72 h the contacts displayed similar *I*–*V* behavior to their original state before plasma treatment (Figure 6c). During this entire period the NWs were stored in UHV and without any photostimulation. These relatively slow transient effects are an important aspect to consider when applying ZnO nanomaterials that require high quality Schottky contacts to maximize performance such as piezoelectric nanogenerators and single NW piezotronic devices.

The degradation of the rectifying properties would appear to be due to rearrangement of surface species and relaxation of higher energy surface states, as the redistribution of dopants or crystallographic point defects in the bulk is unlikely given the lack of external stimuli between measurements and the low applied voltages. XPS of the as-grown samples revealed a high surface carbon concentration for the HoD and LoD samples. Immediately after oxygen plasma treatment, this was significantly reduced but slowly increased in the XPS chamber. The electrical measurements performed after plasma treatment were performed in UHV conditions (10^–11^ mbar) and the carbon growth is expected to be minimal over the measurement period. Therefore, we can expect the surface carbon spectra to be similar to the XPS spectra immediately after plasma treatment. The C 1s peak is known to shift with chemical modification to the carbon layer and also layer thickness, which is evident in the time-lapse XPS data. This is also apparent in the O 1s peak analysis where the small shoulder at 3.4 eV higher BE than O_Zn_ attributed to H_2_O and carbon molecules indicates the adsorption of these species over time in the XPS vacuum chamber. This may help explain the weakening of RR (Figure 6c) and increase in conductivity on the HoDP rectifying contacts that were measured between 24 and 72 h after plasma treatment with donor OH bonds forming on the NW surface and particularly on the defective sloping facets.^17,65^

The difference in resistivity that occurred with oxygen annealing may include a shift in NW Fermi level with fewer carriers after filling of oxygen vacancies, although these as previously mentioned are deep donors. For a surface pinned by adsorbates, a Fermi energy deeper in the bandgap would create greater upward surface band bending. Therefore, the HoD sample most likely has the Fermi level residing closer to the conduction band minimum and a surface balanced by a mixture of donor and acceptor states from adsorbates such as OH, oxygen ions, H_2_O, and carbon molecules. Plasma treatment of HoD NWs results in removal of surface contamination while also removing some OH and filling oxygen vacancies at the surface, resulting in a smaller O_S1_ component. The electrical measurements of the HoDP NWs indicate an increase in free carriers raising the Fermi level or more likely a decrease in surface upward band bending. On exposure to air the surface potential is fixed by adsorbates but with fewer surface defects because of the filling of vacancies. This improves the Schottky contacts by reducing the influence of the defective sloping facets on the contact “edge-effect” and donor defects/species close to the Au interface that affect the transport properties. A similar process occurs with the LoD sample except the annealing reduces the number of free carriers in the bulk and shifts the Fermi level energy deeper in the bandgap while providing fewer bonding sites at the surface. When coupled with the charge trapping nature of adsorbed oxygen surface species from exposure to air a surface region depleted of charge carriers increases the measured resistivity of the NWs. Plasma treatment acts again on the remaining defects particularly at the surface filling vacancies and removing adsorbates creating a potential field at the contact and sloping facets that produces greater current rectification. Therefore, the experimental data points to the overriding effects of surface vacancy defects and adsorbates on the Schottky contacts indicating the influence of the sloping facets on the electrical transport properties in these NWs, due to their defective nature, reactivity, and proximity to the intimate and epitaxial contact interface, cannot be overstated.

## 4 Conclusion

Schottky contacts are sensitive to native point defects, surface states, and interface chemistry. This is none more so apparent in metal contacts with ZnO, particularly oxygen vacancies acting as carrier traps and creating interface states. Here, we have modulated the role of bulk and surface oxygen in ZnO NWs below diameters of 50 nm with Au nanocatalyst contacts and studied the effects on the electrical transport. This shows that through annealing and oxygen plasma the barrier height can be increased on average from 0.3 to 0.5 eV, increasing the rectification by 2 orders of magnitude. Reducing bulk vacancies minimizes tunneling through the depletion region and homogenizing the surface minimizes tunneling at the contact edge. These results show a means to enhance the electrical contacts to NWs for energy harvesting and piezotronic applications, while also confirming the surface processes that can govern Schottky-enhanced piezoelectric and piezotronic NW sensors.

## Supporting Information Available

Example data from the electrical measurements, further STEM data of the crystal polarity, CL plots and corresponding PL data, STEM imaging, XPS data, and EELS/EDX elemental mapping of NWs (PDF)
