# Supplementary material for: Large On–Off Enhancement of Au Nanocatalyst Contacts to ZnO Nanowires with Bulk and Surface Oxygen Modification
Source: ACS Appl Mater Interfaces. 2025 Mar 12;17(12):18996–9011. doi: 10.1021/acsami.4c17872 (PMC11955938; doi:10.1021/acsami.4c17872)
Supplement: Supplementary file 1 — am4c17872_si_001.pdf [file am4c17872_si_001.pdf]

Supporting Information

Large On-Off Enhancement of Au Nanocatalyst Contacts to ZnO Nanowires with Bulk and Surface Oxygen Modification

Alex M. Lord[a,b,*], *Vincent Consonni*[c], *Fabrice Donatini*[d], *Demie M. Kepaptsoglou*[e,f], *Quentin M. Ramasse*[e,g], *Jon E. Evans*[a], *Martin W. Allen*[h], *Mark S'ari*[i, †], *Mac Hathaway*[j], *Irene M. N. Groot*[b]

[a] Centre for NanoHealth, College of Engineering, Swansea University, SA2 8PP, United Kingdom

[b] Leiden Institute of Chemistry, Leiden University, PO Box 9502, Leiden, 2300 RA, Netherlands

[c] CNRS, Grenoble INP, LMGP, Université Grenoble Alpes, F-38000 Grenoble, France

[d] CNRS, Grenoble INP, Institut NEEL, Université Grenoble Alpes, F-38000 Grenoble, France

[e] SuperSTEM Laboratory, SciTech Daresbury Campus, Daresbury, WA4 4AD, United Kingdom.

[f] School of Physics, Engineering and Technology, University of York, Heslington, York, YO10 5DD, United Kingdom

[g] School of Chemical and Process Engineering and School of Physics and Astronomy, University of Leeds, Leeds, LS2 9JT, United Kingdom

[h] MacDiarmid Institute for Advanced Materials and Nanotechnology, Department of Electrical and Computer Engineering, University of Canterbury, Christchurch 8140, New Zealand

[i] School of Chemical and Process Engineering, University of Leeds, Leeds, LS2 9JT, United Kingdom

[j] Harvard Center for Nanoscale Systems, Cambridge, Massachusetts 02138, United States of America

*Corresponding Author - E-mail address: a.m.lord@lic.leidenuniv.nl

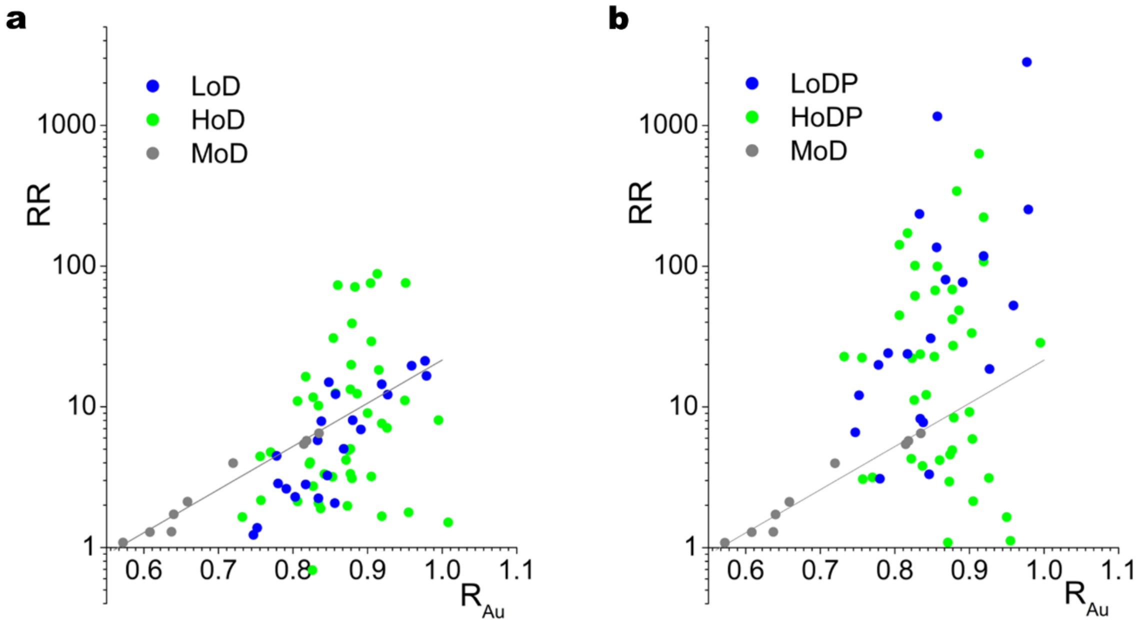

Figure S1. The same data as shown in Figure 1 of the main manuscript but without error bars for the nanowires, (a) before, and (b) after oxygen plasma treatment.

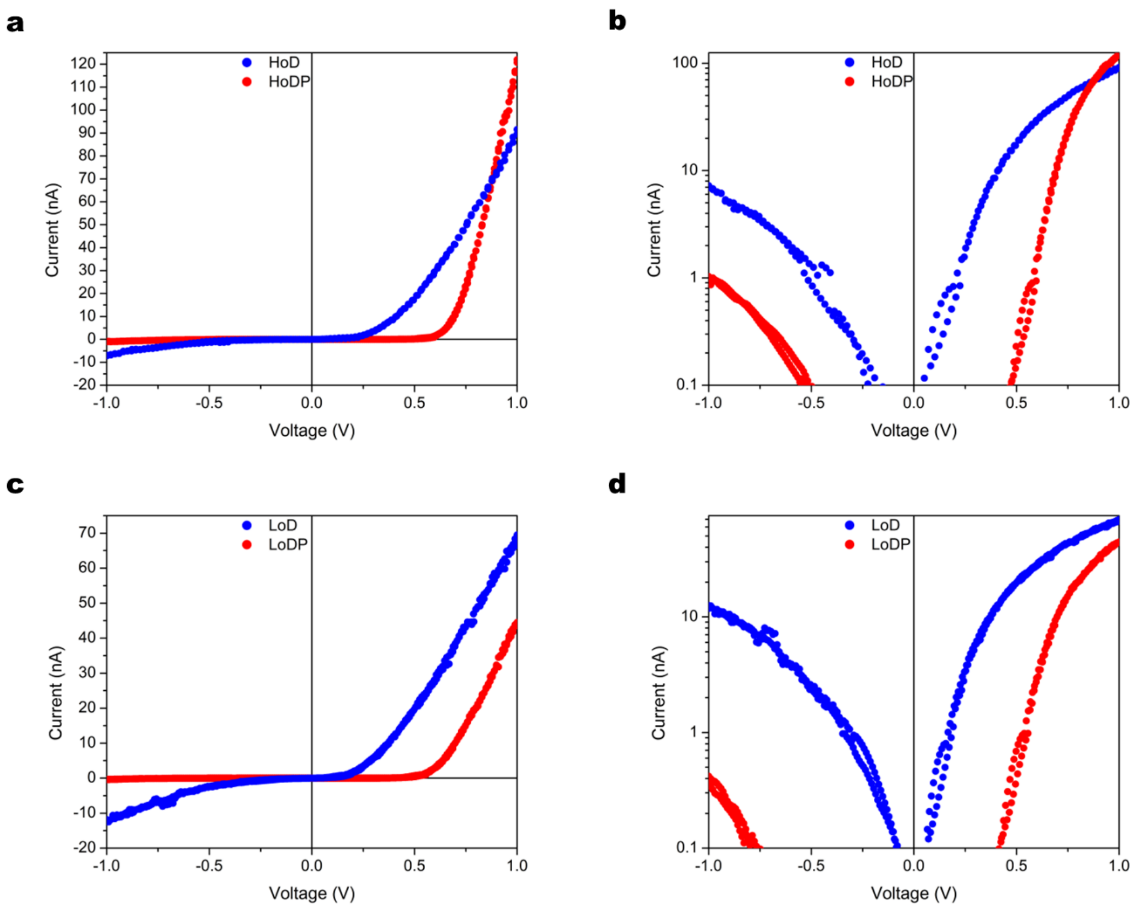

Figure S2. (a) and (b) show the I-V data for a HoD NW (blue) and the same NW after plasma treatment HoDP (red) in linear and log-linear format. Similarly, (c) and (d) show the data for a LoD (blue) NW before and after plasma treatment LoDP (red).

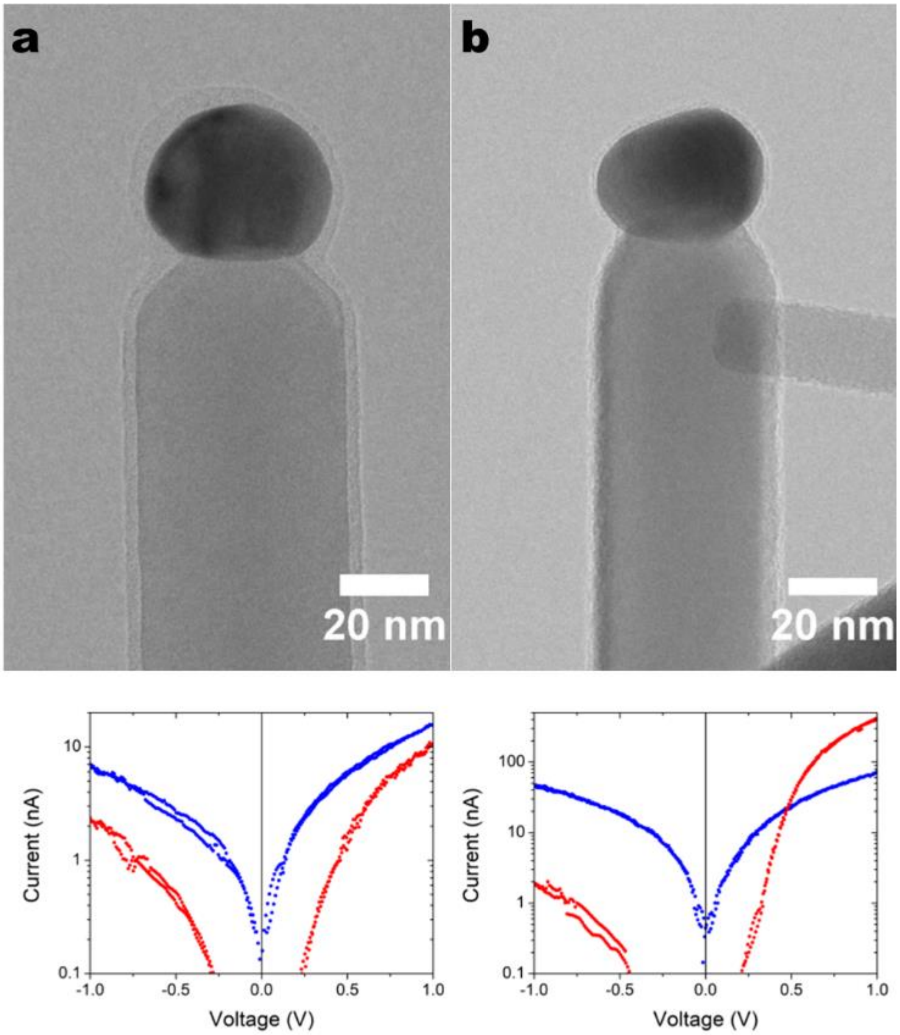

Figure S3. Bright field images of (a) a LoDP nanowire, and (b) a HoDP nanowire after oxygen plasma treatment and electrical measurement. The corresponding I-V below each image shows the I-V data before (blue) and after (red) oxygen plasma treatment.

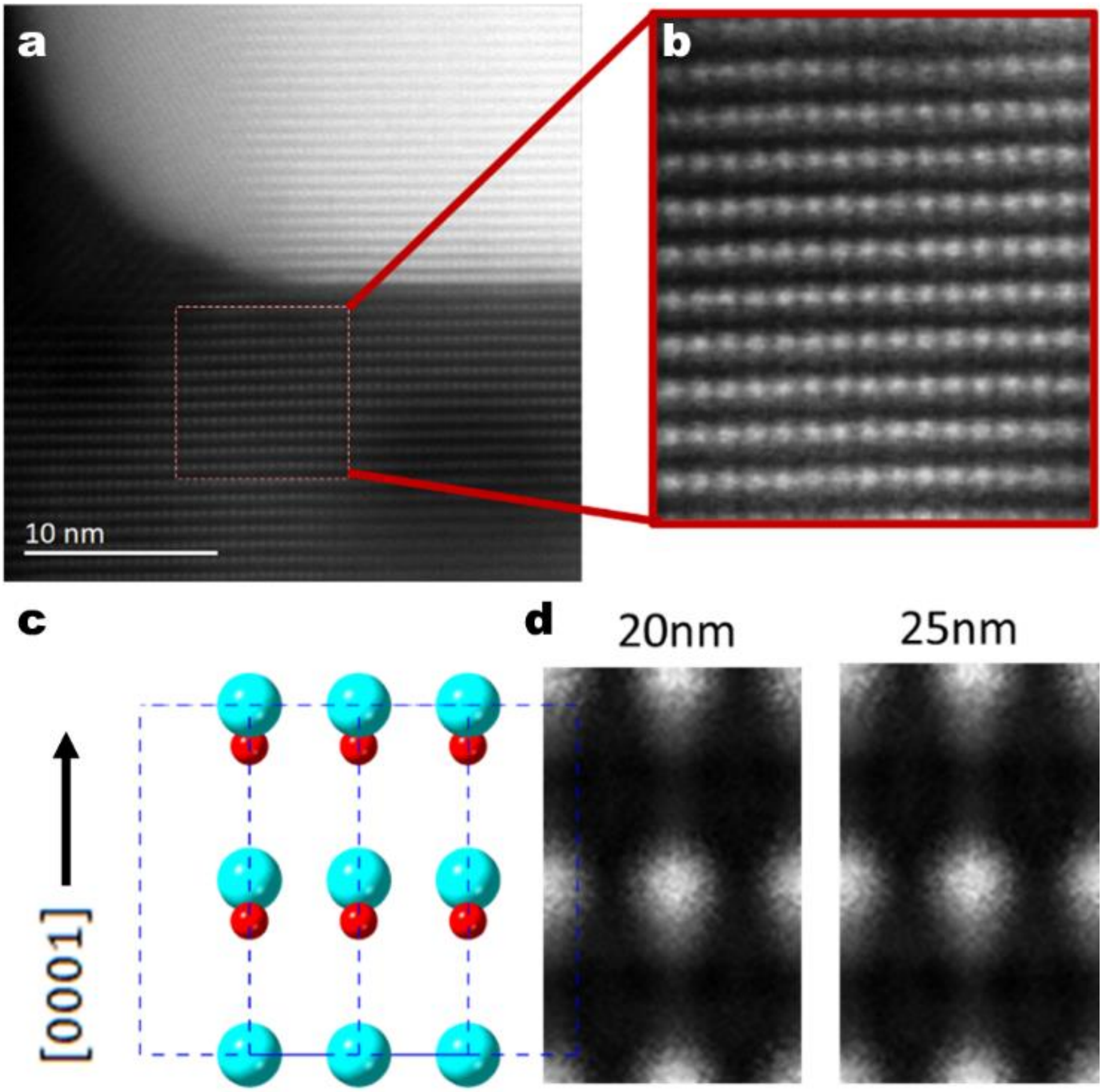

Figure S4. HAADF imaging analysis to determine ZnO polarity. (a) Atomic-resolution HAADF image of the near-interface region of the nanowire located on the $[01\bar{1}0]$ zone axis showing the Zn columns and Au catalyst particle lattice. (b) High magnification HAADF image of the same region showing the Zn atomic columns (bright dots) and a weaker signal below each Zn column. (c) 'ball-and-stick' model depicting the atomic arrangement of Zn (blue) and O (red) columns when viewed on the $[01\bar{1}0]$ zone axis. (d) HAADF simulations of a ZnO slab of 20nm and 25nm thicknesses replicating the bright Zn columns and weaker O atomic columns located below the Zn columns as seen in (b).

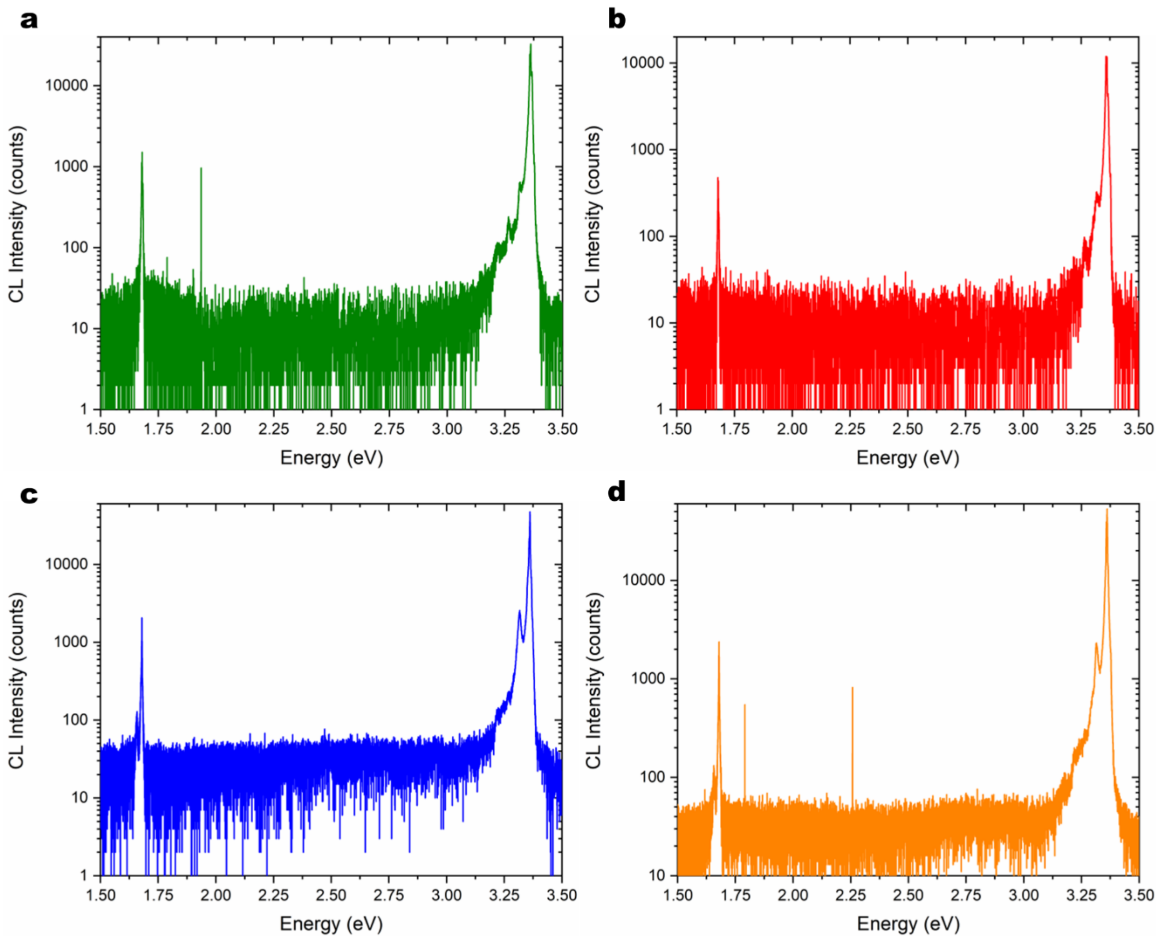

Figure S5. High resolution 5k CL spectra of the visible and near band edge region for each sample: (a) HoD ; (b) LoD; (c) HoDP; (d) LoDP.

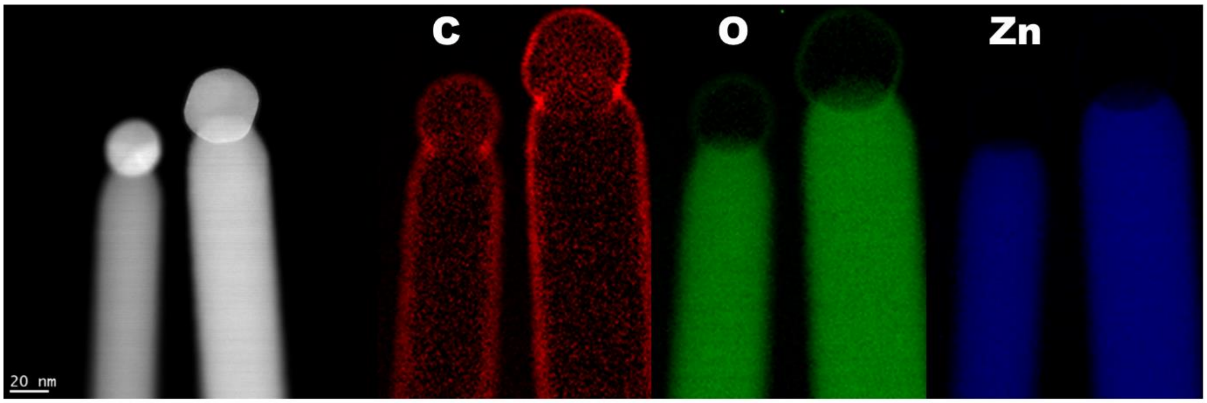

Figure S6. MAADF image of two LoDP nanowires after electrical measurement and then exposed to atmosphere for several days before STEM imaging and the corresponding EELS maps of C, O, and Zn.

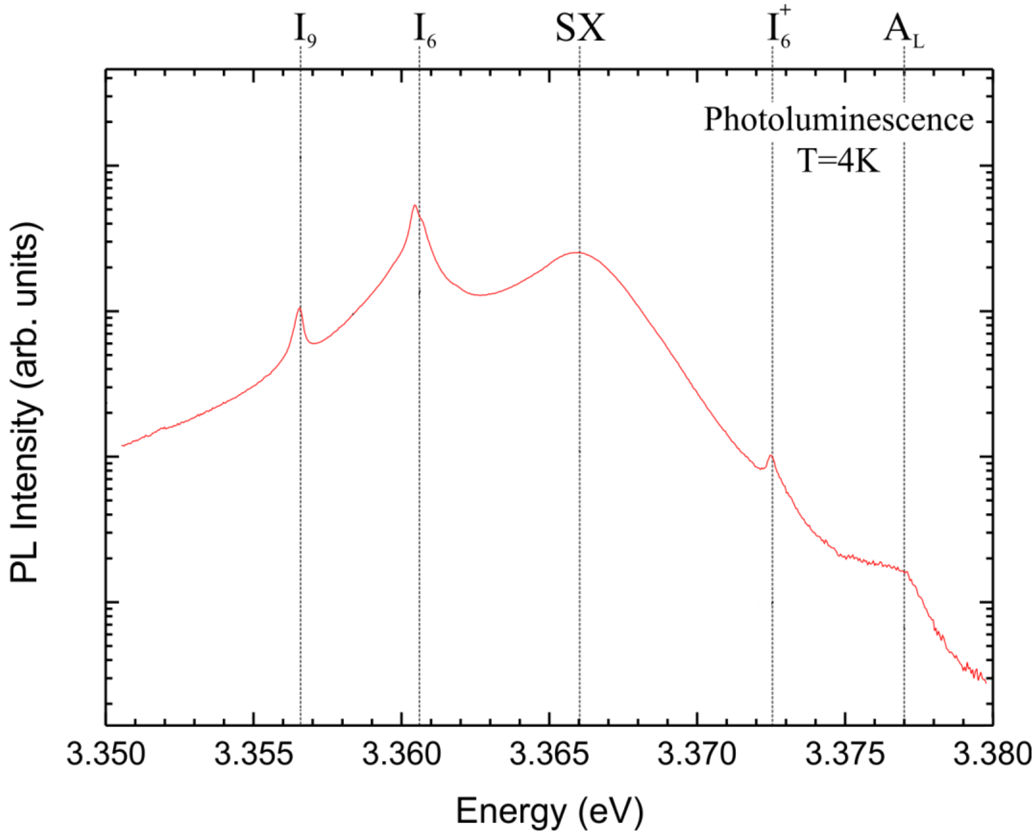

Figure S7. Photoluminescence spectroscopy of an unmodified nanowire array performed at 4 K.

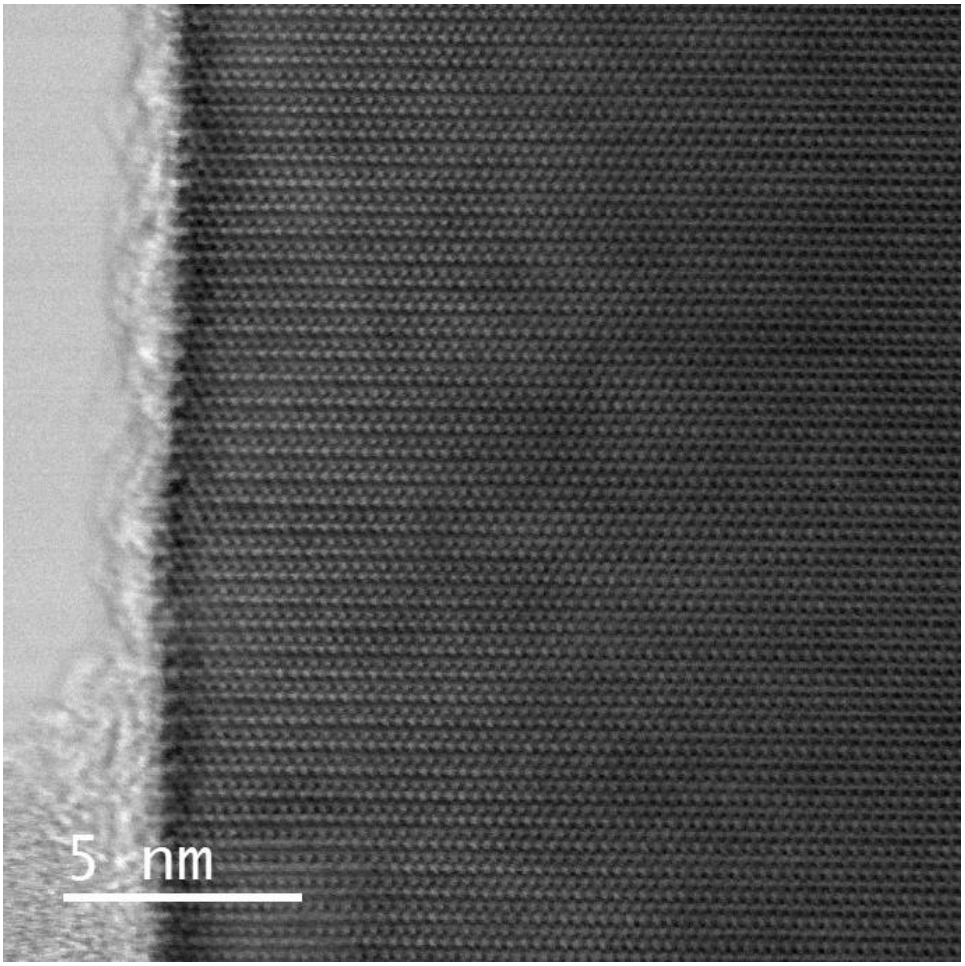

Figure S8. Atomic-resolution bright-field STEM image of an LoDP NW showing the atomic smoothness and regularity of the surface after the plasma treatment.

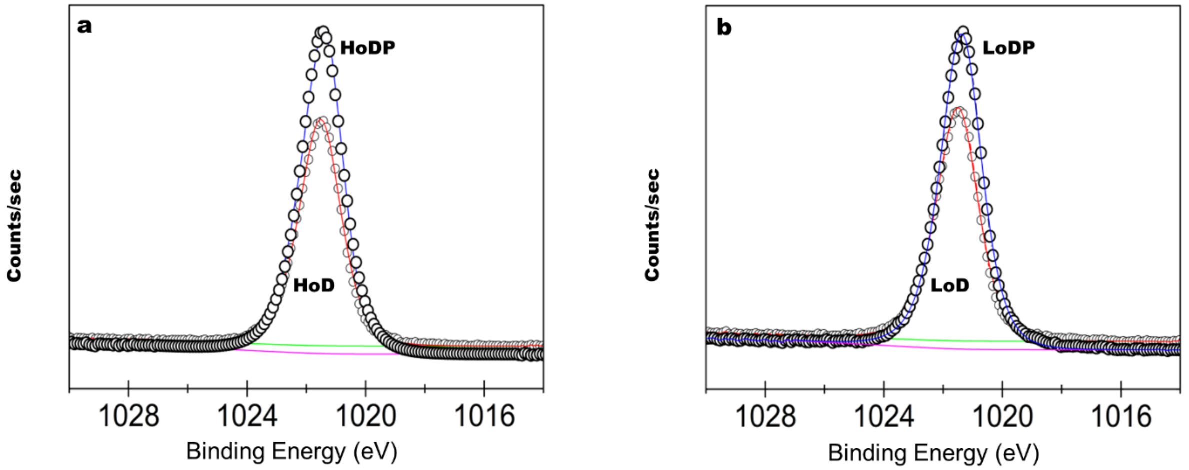

Figure S9. XPS data (black circles) of the Zn 2p3/2 peak including the single component fit to match the envelope to the experimental data comprising of samples (a) HoD (red) and HoDP (blue) and (b) LoD (red) and LoDP (blue) showing the difference in the raw data before and after oxygen plasma treatment of the HoD and LoD samples.

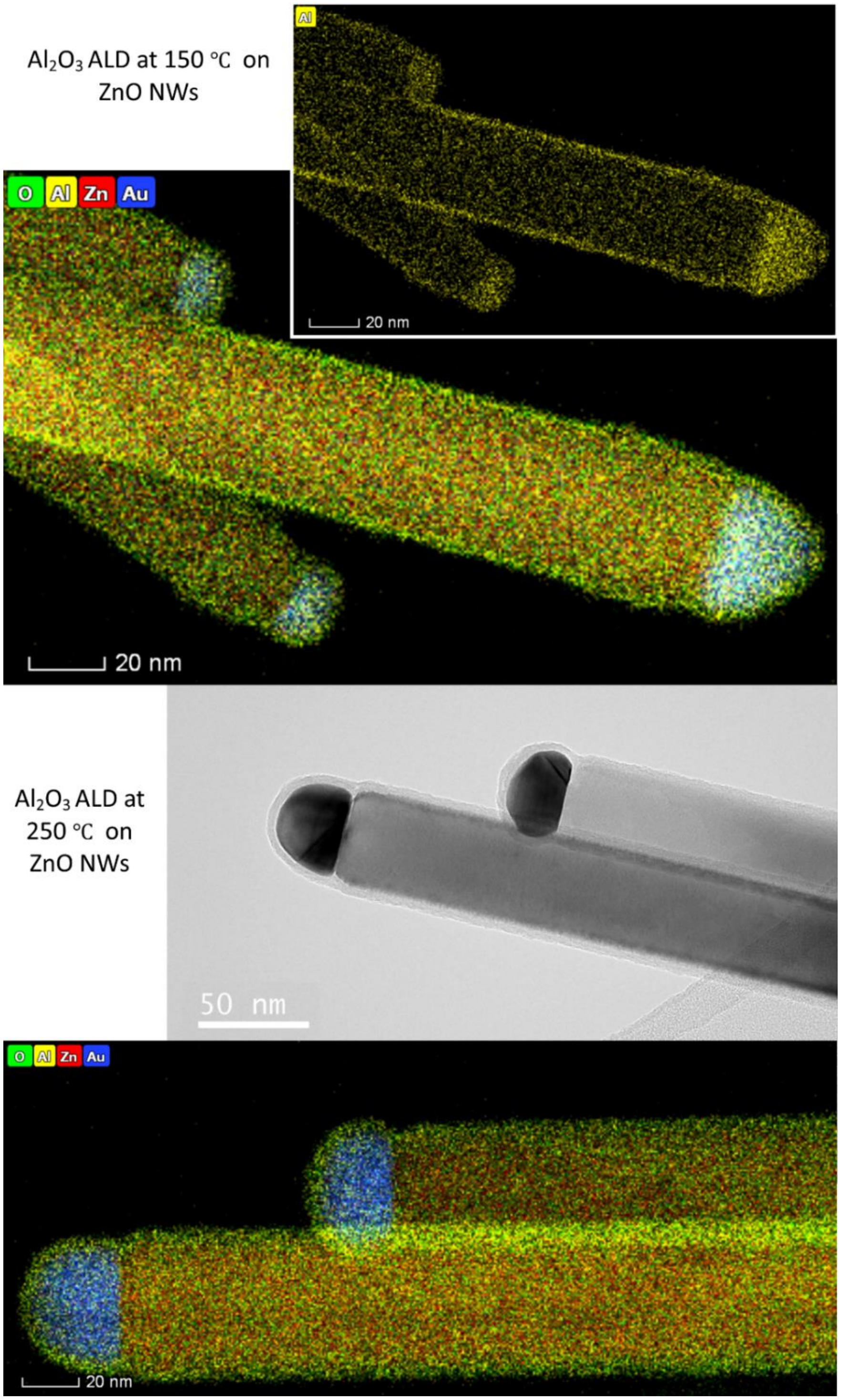

Figure S10. TEM-EDX images of Al_2O_3 deposited by atomic-layer deposition on a Savannah 200 ALD system with 30 cycles at 150 ℃ (~2.5 nm thick) and 250 ℃ (~3.5 nm thick) showing the lower temperature produces a thinner oxide passivation layer.
